# A weighted average difference method for detecting differentially expressed genes from microarray data

**DOI:** 10.1186/1748-7188-3-8

**Published:** 2008-06-26

**Authors:** Koji Kadota, Yuji Nakai, Kentaro Shimizu

**Affiliations:** 1 Graduate School of Agricultural and Life Sciences, The University of Tokyo, 1-1-1 Yayoi, Bunkyo-ku, Tokyo 113-8657, Japan

## Abstract

**Background:**

Identification of differentially expressed genes (DEGs) under different experimental conditions is an important task in many microarray studies. However, choosing which method to use for a particular application is problematic because its performance depends on the evaluation metric, the dataset, and so on. In addition, when using the Affymetrix GeneChip^® ^system, researchers must select a preprocessing algorithm from a number of competing algorithms such as MAS, RMA, and DFW, for obtaining expression-level measurements. To achieve optimal performance for detecting DEGs, a suitable combination of gene selection method and preprocessing algorithm needs to be selected for a given probe-level dataset.

**Results:**

We introduce a new fold-change (FC)-based method, the weighted average difference method (WAD), for ranking DEGs. It uses the average difference and relative average signal intensity so that highly expressed genes are highly ranked on the average for the different conditions. The idea is based on our observation that known or potential marker genes (or proteins) tend to have high expression levels. We compared WAD with seven other methods; average difference (AD), FC, rank products (RP), moderated *t *statistic (modT), significance analysis of microarrays (samT), shrinkage *t *statistic (shrinkT), and intensity-based moderated *t *statistic (ibmT). The evaluation was performed using a total of 38 different binary (two-class) probe-level datasets: two artificial "spike-in" datasets and 36 real experimental datasets. The results indicate that WAD outperforms the other methods when sensitivity and specificity are considered simultaneously: the area under the receiver operating characteristic curve for WAD was the highest on average for the 38 datasets. The gene ranking for WAD was also the most consistent when subsets of top-ranked genes produced from three different preprocessed data (MAS, RMA, and DFW) were compared. Overall, WAD performed the best for MAS-preprocessed data and the FC-based methods (AD, WAD, FC, or RP) performed well for RMA and DFW-preprocessed data.

**Conclusion:**

WAD is a promising alternative to existing methods for ranking DEGs with two classes. Its high performance should increase researchers' confidence in microarray analyses.

## Background

One of the most common reasons for analyzing microarray data is to identify differentially expressed genes (DEGs) under two different conditions, such as cancerous versus normal tissue [1]. Numerous methods have been proposed for doing this [2-27], and several evaluation studies have been reported [28-32]. A prevalent approach to such an analysis is to calculate a statistic (such as the *t*-statistic or the fold change) for each gene and to rank the genes in accordance with the calculated values (*e.g*., the method of Tusher et al. [3]). A large absolute value is evidence of a differential expression. Inevitably, different methods (statistics) generally produce different gene rankings, and researchers have been troubled about the differences. Another approach is to rank genes in accordance with their predictive accuracy such as by performing gene-by-gene prediction [24].

Although the two approaches are not mutually exclusive, their suitabilities differ; the former approach is better when the identified DEGs are to be investigated for a follow-up study [24], and the latter is better when a classifier or predictive model needs to be developed for class prediction [17]. The method presented in this paper focuses on the former approach – many "wet" researchers want to rank the true DEGs as high as possible, and the former approach is more suitable for that purpose.

Methods for ranking genes in accordance with their degrees of differential expression can be divided into *t*-statistic-based methods and fold-change (FC)-based methods. Both types are commonly used for selecting DEGs with two classes. They each have certain disadvantages. The *t*-statistic-based gene ranking is deficient because a gene with a small fold change can have a very large statistic for ranking, due to the *t*-statistic possibly having a very small denominator [24]. The FC-based ranking is deficient because a gene with larger variances has a higher probability of having a larger statistic [24]. From our experience, a disadvantage that they share is that some top-ranked genes which are falsely detected as "differentially expressed" tend to exhibit lower expression levels. This interferes with the chance of detecting the "true" DEGs because the relative error is higher at lower signal intensities [4,33-36]. Although many researchers have addressed this problem, false positives remain to some extent in the subset of top-ranked genes.

Our weighted average difference (WAD) method was designed for accurate gene ranking. We evaluated its performance in comparison with those of the average difference (AD) method, the FC method, the rank products (RP) method [12,37], the moderated *t *statistic (modT) method [9], the significance analysis of microarrays *t *statistic (samT) method [3], the shrinkage *t *statistic (shrinkT) method [23], and the intensity-based moderated *t *statistic (ibmT) method [20] by using datasets with known DEGs (Affymetrix spike-in datasets and datasets containing experimentally validated DEGs).

## Results and discussion

The evaluation was mainly based on the area under the receiver operating characteristic (ROC) curve (AUC). The AUC enables comparisons without a trade-off in sensitivity and specificity because the ROC curve is created by plotting the true positive (TP) rate (sensitivity) against the false positive (FP) rate (1 minus the specificity) obtained at each possible threshold value [38-40]. This is one of the most important characteristics of a method. The evaluation was performed using 38 different datasets [41-73] containing true DEGs that enabled us to determine the TP and FP.

Seven methods were used for comparison: AD was used to evaluate the effect of the "weight" term in WAD (see the Methods section), FC was recommended by Shi et al. [74], RP [12] and modT [9] were recommended by Jeffery et al. [29], samT [3] is a widely used method, and shrinkT [23] and ibmT [20] were recently proposed at the time of writing. All programming was done in R [75] using Bioconductor [76].

### Datasets

The evaluation used two publicly available spike-in datasets [41,42] (Datasets 1 and 2) and 36 experimental datasets that each had some true DEGs confirmed by real-time polymerase chain reaction (RT-PCR) [43-73] (Dataset 3–38). The first two datasets are well-chosen sets of data from other studies [20,23]. Dataset 1 is a subset of the completely controlled Affymetrix spike-in study done on the HG-U95A array [41], which contains 12,626 probesets, 12 technical replicates of two different states of samples, and 16 known DEGs. The details of this experiment are described elsewhere [41]. The subset was extracted from the original sets by following the recommendations of Opgen-Rhein and Strimmer [23]. Dataset 2 was produced from the Affymetrix HG-U133A array, which contains 22,300 probesets, three technical replicates of 14 different states of samples, and 42 known DEGs. Accordingly, there were 91 possible comparisons (_14_C_2 _= 91). Dataset 2 was evaluated on the basis of the average values of the 91 results.

Since these experiments (using Datasets 1 and 2) were performed using the Affymetrix GeneChip^® ^system, one of several available preprocessing algorithms (such as Affymetrix Microarray Suite version 5.0 (MAS) [77], robust multichip average (RMA) [38], and distribution free weighted method (DFW) [40]) could be applied to the probe-level data (.CEL files). We used these three algorithms to preprocess the probe-level data; MAS and RMA are most often used for this purpose, and DFW is currently the best algorithm [40]. Of these, DFW is essentially a summarization method and its original implementation consists of following steps: no background correction, quantile normalization (same as in RMA), and DFW summarization. The probeset summary scores for Datasets 1 and 2 are publicly available on-line [42]. Accordingly, a total of six datasets were produced from Datasets 1 and 2, *i.e*., Dataset *x *(MAS), Dataset *x *(RMA), Dataset *x *(DFW), where *x *= 1 or 2.

Datasets 3–38 were produced from the Affymetrix HG-U133A array, which is currently the most used platform. All of the datasets consisted of two different states of samples (*e.g*., cancerous vs. non-cancerous) and the number of samples in each state was > = 3. Each dataset had two or more true DEGs and these DEGs were originally detected on MAS- or RMA-preprocessed data. The raw (probe-level) data are also publicly available from the Gene Expression Omnibus (GEO) website [78]. One can preprocess the raw data using the MAS, RMA, and DFW algorithms. Detailed information on these datasets is given in the additional file [see Additional file 1].

### Evaluation using spike-in datasets (Datasets 1 and 2)

The AUC values for the eight methods for Datasets 1 and 2 are shown in Table 1. Overall, WAD outperformed the other methods. It performed the best for five of the six datasets and ranked no lower than fourth best for all datasets. RP performed the best for Dataset 2 (RMA). The R-codes for analyzing these datasets are available in the additional files [see Additional files 2 and 3].

**Table 1:** AUC (percent) values for Datasets 1 and 2 for eight methods

	MAS		RMA		DFW	
			
Method	Dataset 1	Dataset 2	Dataset 1	Dataset 2	Dataset 1	Dataset 2
WAD	**96.772(1)**	**97.684(1)**	**99.980(1)**	98.240(4)	**100.00(1)**	**99.953(1)**
AD	83.381(6)	96.430(8)	99.897(6)	98.631(2)	**100.00(1)**	99.948(2)
FC	83.092(7)	96.445(7)	99.655(8)	98.617(3)	**100.00(1)**	99.948(2)
RP	81.981(8)	96.626(6)	99.757(7)	**99.161(1)**	99.993(4)	99.938(3)
modT	93.257(4)	97.561(4)	99.928(5)	98.109(7)	99.983(7)	98.459(6)
samT	94.002(3)	97.547(5)	99.944(3)	98.139(6)	99.988(5)	98.656(4)
shrinkT	92.379(5)	97.617(3)	99.955(2)	97.846(8)	99.984(6)	98.558(5)
ibmT	94.693(2)	97.618(2)	99.941(4)	98.183(5)	99.983(7)	98.455(7)

Numbers in parentheses show the rankings. Signal intensities smaller than 1 in the MAS-preprocessed data were set to 1 so that the logarithm of the data could be taken.

The largest difference between WAD and the other methods was observed for Dataset 1 (MAS). Because MAS uses local background subtraction, MAS-preprocessed data tend to have extreme variances at low intensities. As shown in Table 2, increasing the floor values for the MAS-preprocessed data increased the AUC values for all methods except WAD. Nevertheless, the AUC values for WAD at the four intensity thresholds were clearly higher than those for the other methods. These results indicate that the advantage of WAD over the other methods is not merely due to a defect in the MAS algorithm.

**Table 2:** AUC (percent) values for Dataset 1 (MAS) for different signal intensity thresholds

	Signal intensity threshold			
	
Method	1	5	10	15
WAD	**96.772(1)**	**99.052(1)**	**99.228(1)**	**98.506(1)**
AD	83.381(6)	89.215(6)	92.996(7)	94.915(6)
FC	83.092(7)	88.353(8)	92.381(8)	94.455(7)
RP	81.981(8)	88.516(7)	93.131(6)	95.456(5)
modT	93.257(4)	94.776(2)	95.284(3)	95.977(4)
samT	94.002(3)	94.731(4)	95.074(5)	96.028(3)
shrinkT	92.379(5)	94.114(5)	95.537(2)	96.437(2)
ibmT	94.693(2)	94.770(3)	95.260(4)	94.318(8)

AUC values when floor signal values in MAS-preprocessed data were 1, 5, 10, and 15, corresponding to substitutions of 4.1, 24.7, 40.2, and 50.8% of signals.

The basic assumption of WAD is that "strong signals are better signals." This assumption may unfairly favorable when spike-in datasets are used for evaluation. One can only spike mRNA at rather high concentrations because of technical limitations such as mRNA stability and pipetting accuracy, meaning that spike-in transcripts tend to have strong signals [79]. The basic assumption is therefore necessarily true for spike-in data. Indeed, a statistic based on the relative average signal intensity (*e.g*., a statistic based on the "weight" term, *w*, in the WAD statistic; see Methods) for Dataset 1 (MAS) could, for example, give a very high AUC value of 90.0%. We also observed high AUC values based on the *w *statistic for the RMA- (87.3% of AUC) and DFW-preprocessed data (80.4%).

### Evaluation using experimental datasets (Datasets 3–38)

**Figure 1:**
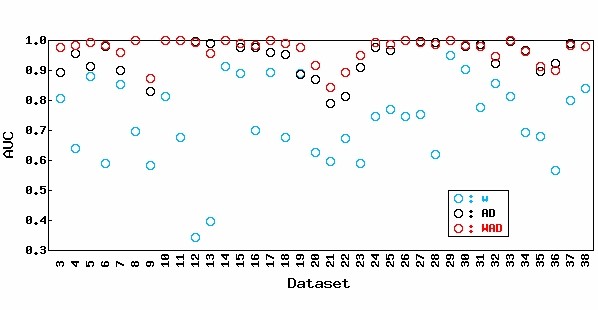
**Effect of the weight (*w*) term in WAD statistic for 36 real experimental datasets (Datasets 3–38)**. AUC values for the weight term (*w*, light blue circle) in WAD, AD (black circle), and WAD (red circle) are shown. Analyses of Datasets 3–26 and Datasets 27–38 were performed using MAS- and RMA-preprocessed data, respectively, following the choice of preprocessing algorithm in the original papers. The average AUC values for their respective methods as well as the other methods are shown in Table 3. Note that WAD statistics (AD with the *w *term) can overall give higher AUC values than AD statistics.

Nevertheless, we have seen that several well-known marker genes and experimentally validated DEGs tend to have strong signals, which supports our basic assumption. If there is no correlation between differential expression and expression level, the AUC value based on the *w *statistic should be approximately 0.5. Actually, of the 36 experimental datasets, 34 had AUC values > 0.5 when the *w *statistic was used (Figure 1, light blue circle) and the average AUC value was high (72.7%). These results demonstrate the validity of our assumption.

This high AUC value may not be due to the microarray technology because any technology is unreliable at the low intensity/expression end. Inevitably, genes that can be confirmed as DEGs using a particular technology tend to have high signal intensity. That is, it is difficult to confirm candidate genes having low signal intensity [48,80]. Whether a candidate is a true DEG must ultimately be decided subjectively. Therefore, many candidates having low signal intensity should not be considered true DEGs.

Apart from the above discussion, a good method should produce high AUC values for real experimental datasets. The analysis of Datasets 3–38 showed that the average AUC value for WAD (96.737%) was the highest of the eight methods when the preprocessing algorithms were selected following the original studies (Table 3). WAD performed the best for 12 of the 36 experimental datasets.

**Table 3:** Results for Dataset 3–38 using eight methods

Method	Average AUC (%)	No. of datasets best performed
WAD	**96.737 **	**12**
AD	94.758	1
FC	94.659	4
RP	93.182	2
modT	95.541	1
samT	95.866	7
shrinkT	95.439	4
ibmT	96.060	5

Analyses of Datasets 3–26 and Datasets 27–38 were performed using MAS- and RMA-preprocessed data, respectively, following the choice of preprocessing algorithm in the original papers. Accordingly, the average AUC value was calculated from those for Datasets 3–26 (MAS) and Datasets 27–38 (RMA). The best performing methods for each dataset is given in the additional file [see Additional file 1].

The 36 experimental datasets can be divided into two groups: One group (Datasets 3–26) had originally been analyzed using MAS-preprocessed data and the other (Datasets 27–38) had originally been analyzed using RMA-preprocessed data. Table 4 shows the average AUC values for MAS-, RMA-, and DFW-preprocessed data for the two groups (Datasets 3–26 and Datasets 27–38). The values for the MAS- (RMA-) preprocessed data for the first (second) group were overall the best among the three preprocessing algorithms. This is reasonable because the best performing algorithms were practically used in the original papers [43-73]. The exception was for RP [12] in the first group: the average AUC values for RMA- (92.540) and DFW-preprocessed data (92.534) were higher than the value for MAS-preprocessed data (91.511).

**Table 4:** Average AUC values for Datasets 3–26 and 27–38

	Datasets 3–26			Datasets 27–38			
			
Method	MAS	RMA	DFW	MAS	RMA	DFW	Average
WAD	**96.740(1)**	91.373(6)	91.407(5)	**92.416(1)**	96.732(2)	94.090(4)	**93.793 **
AD	93.755(6)	93.098(2)	92.239(2)	87.411(7)	**96.766(1)**	94.222(2)	92.915
FC	93.625(7)	**93.117(1)**	92.239(2)	88.230(6)	96.726(3)	94.221(3)	93.026
RP	91.511(8)	92.540(3)	**92.534(1)**	84.552(8)	96.526(4)	**94.665(1)**	92.055
modT	95.673(5)	91.381(5)	90.109(7)	90.895(4)	95.277(7)	92.355(7)	92.615
samT	95.947(3)	91.231(8)	89.959(8)	90.305(5)	95.702(5)	92.052(8)	92.533
shrinkT	95.733(4)	91.316(7)	91.451(4)	90.968(3)	94.851(8)	93.684(5)	93.001
ibmT	96.344(2)	91.771(4)	90.252(6)	91.921(2)	95.491(6)	92.427(6)	93.034

Average	94.916	91.978	91.274	89.587	96.009	93.465	

Analyses of Datasets 3–26 and Datasets 27–38 were performed using MAS- and RMA-preprocessed data, respectively, following the choice of preprocessing algorithm in the original papers.

Interestingly, the FC-based methods (AD, WAD, FC, and RP) were generally superior to the *t*-statistic-based methods (modT, samT, shrinkT, and ibmT) when RMA- or DFW-preprocessed data were analyzed. This is probably because the RMA and DFW algorithms simultaneously preprocess data across a set of arrays to improve the precision of the final measures of expression [81] and include a variance stabilization step [38,40]. Accordingly, some variance estimation strategies employed in the *t*-statistic-based methods may be no longer necessary for such preprocessed data. Indeed, the *t*-statistic-based methods were clearly superior to the FC-based methods (except WAD) when the MAS-preprocessed data were analyzed: The MAS algorithm considers data on a per-array basis [77] and has been criticized for its exaggerated variance at low intensities [82].

It should be noted that we cannot compare the three preprocessing algorithms with the results from the 36 real experimental datasets. One might think the RMA algorithm is the best among the three algorithms because (1) the average AUC values for the RMA (the average is 91.978) were higher than those for DFW (91.274) in the results for Datasets 3–26 and (2) the average AUC values for DFW (93.465) were also higher than those for MAS (89.587) in the results for Datasets 27–38 (Table 4). However, the lower average AUC values for DFW compared with the RMA in the results for Datasets 3–26 were mainly due to the poor affinity between the *t*-statistic-based methods and the DFW algorithm. The average AUC values for DFW were quite similar to those for RMA only when the FC-based methods were compared. In addition, the higher average AUC values for DFW (93.465) than for MAS in the results for Datasets 27–38 were rather by virtue of the similarity of data processing to RMA: DFW employs the same background correction and normalization procedures as RMA, and the only difference between the two algorithms is in their summarization procedure.

It should also be noted that there must be many additional DEGs in the 36 experimental datasets because the RT-PCR validation is performed only for a subset of top-ranked genes. Accordingly, we cannot compare the eight methods by using other evaluation metrics such as the false discovery rate (FDR) [83] or compare their abilities of identifying new genes that might have been missed in a previous analysis. Such comparisons could also produce different results with different parameters such as number of top ranked genes or different gene ranking methods used in the original study. For example, the FC-based methods (AD, WAD, FC, and RP) and the *t*-statistic-based methods (modT, samT, shrinkT, and ibmT) produce clearly dissimilar gene lists (see Table 5). This difference suggests that the FC-based methods should be advantageous for six datasets (Datasets 3–6 and 27–28) whose gene rankings were originally performed with only the FC-based methods. Likewise, the *t*-statistic-based methods should be advantageous for 15 datasets (Datasets 19–26 and 32–38). The RT-PCR validation for a subset of potential DEGs were based on those gene ranking results. Indeed, the average rank (3.92) of AUC values for the FC-based methods on the six datasets and for the *t*-statistic-based methods on the 15 datasets was clearly higher than that (5.08) for the *t*-statistic-based methods on the six datasets and for the FC-based methods on the 15 datasets (*p*-value = 0.001, Mann-Whitney *U *test). This implies a comparison using a total of the 21 datasets (Datasets 3–6, 19–28, and 32–38) should give an advantageous result for the *t*-statistic-based methods since those methods were used in the original analysis for 15 of the 21 datasets. Nevertheless, the best performing methods across the 36 experimental datasets including the 21 datasets seem to be independent of the originally analyzed methods, by virtue of WAD's high performance. Also, the overall performances of eight methods for the two artificial spike-in datasets (Datasets 1 and 2) and for the 36 real experimental datasets (Datasets 3–38) were quite similar (Tables 1 and 4). These results suggest that the use of genes only validated by RT-PCR as DEGs does not affect the objective evaluations of the methods.

**Table 5:** Average number of genes common to each pair of methods for Datasets 3–38

(a) MAS	AD	FC	RP	modT	samT	shrinkT	ibmT
WAD	52.0	39.1	49.7	37.7	45.2	39.8	42.8
AD		61.9	84.1	34.4	47.1	37.2	33.2
FC			58.2	29.5	39.1	31.2	28.1
RP				30.5	41.8	32.4	29.8
modT					79.9	92.7	78.1
samT						83.5	65.0
shrinkT							74.8

(b) RMA	AD	FC	RP	modT	samT	shrinkT	ibmT

WAD	62.2	50.4	60.2	31.7	32.6	30.8	33.4
AD		78.8	84.7	35.2	36.2	33.9	38.0
FC			72.3	32.2	32.8	30.8	34.5
RP				36.8	37.6	35.4	39.4
modT					88.3	93.0	88.4
samT						87.6	83.6
shrinkT							85.1

(c) DFW	AD	FC	RP	modT	samT	shrinkT	ibmT

WAD	84.3	83.9	72.1	13.6	13.4	14.6	13.9
AD		98.6	77.3	13.7	13.5	14.7	14.0
FC			77.0	13.6	13.4	14.6	13.9
RP				18.1	17.9	20.1	18.8
modT					94.1	83.2	93.4
samT						81.1	91.0
shrinkT							83.0

The averages were calculated from top 100 genes. Due to the symmetric nature of the matrix only the upper triangular part is presented.

To our knowledge, the number (32) of real experimental datasets we analyzed is much larger than those analyzed by previous methodological studies: Two experimental datasets were evaluated for the ibmT [20] method and one was for the shrinkT [23] method. Although those studies performed a profound analysis on a few datasets, we think a superficial comparison on a large number of experimental datasets is more important than a profound one on a few experimental datasets when estimating the methods' practical ability to detect DEGs, as the superficial comparison on a large number of datasets can also prevent selection bias regarding the datasets. Therefore, we think the number of experimental datasets interrogated is also very important for evaluating the practical advantages of the existing methods. A profound comparison on a large number of experimental datasets should be of course the most important. For example, a comparison of significant Gene Ontology [84] categories using top-ranked genes from each of the eight methods would be interesting. We think such a comparison would be important as another reasonable assessment of whether some top-ranked genes detected only by WAD might actually be differentially expressed. The analysis of many datasets is however practically difficult because of wide range of knowledge it would require, and this related to the next task.

### Effect of different preprocessing algorithms on gene ranking

In general, different choices of preprocessing algorithms can output different subsets of top-ranked genes (*e.g*., see Tables 1 and 4) [85]. We compared the gene rankings of MAS-, RMA-, and DFW-preprocessed data. Table 6 shows the average number of common genes in 20, 50, 100, and 200 top-ranked genes for the 36 experimental datasets. Although all methods output relatively low numbers of common genes, the numbers for WAD were consistently higher than those for the other methods. This result indicates the gene ranking based on WAD is more robust against data processing than the other methods are.

**Table 6:** Average number of common genes in results of three preprocessing algorithms for Datasets 3–38

Method	Top 20	Top 50	Top 100	Top 200
WAD	**8.2 **	**19.8 **	**38.0 **	**73.1 **
AD	4.5	10.7	20.0	37.9
FC	5.0	12.2	22.0	40.6
RP	4.6	11.1	20.6	40.5
modT	4.4	13.1	27.6	60.3
samT	4.0	11.9	24.4	52.4
shrinkT	4.5	13.6	29.0	62.4
ibmT	5.3	15.2	32.0	66.7

From the comparison of WAD and AD, it is obvious that the high rank-invariant property of WAD is by virtue of the inclusion of the weight term: The gene ranking based on the *w *statistic is much more reproducible than the one based on the AD statistic. Relatively small numbers of common genes were observed for the other FC-based methods (AD, FC, and RP) (Table 6). This was because differences in top-ranked genes between MAS and RMA (or DFW) were much larger than those between RMA and DFW (data not shown).

### Effect of outliers on the weight term in WAD statistic

Recall that the WAD statistic is composed of the AD statistic and the weight (*w*) term (see the Methods section). Some researchers may be suspicious about the use of *w *because it is calculated from a sample mean (*i.e*., $\bar{x_{i}}$) for gene *i*, and sample means are notoriously sensitive to outliers in the data. Actually, the *w *term is calculated from logged data and is therefore insensitive to outliers. Indeed, we observed few outliers in two datasets (there were 31 outliers in Dataset 14 and 7 outliers in Dataset 29; they corresponded to (31 + 7)/(22,283 clones × 36 datasets) = 0.0047%) when an outlier detection method based on Akaike's Information Criterion (AIC) [85-87] was applied to the average expression vector $\left(\bar{x_{1}},...,\bar{x_{p}}\right)$ calculated from each of the 36 datasets. In addition to the automatic detection of outliers, we also visually examined the distribution of the average vectors and concluded there were no outliers. Also, the differences in the AUC values between AD and WAD were less than 0.1% for the two datasets (Datasets 14 and 29). We therefore decided that all the automatically detected outliers did not affect the result. The average expression vectors and the results of outlier detection using the AIC-based method are available in the additional files [see Additional files 4 and 5].

### Choice of best methods with preprocessing algorithms

In this study, we analyzed eight gene-ranking methods with three preprocessing algorithms. Currently, there is no convincing rationale for choosing among different preprocessing algorithms. Although the three algorithms from best to worst were DFW, RMA, and MAS when artificial spike-in datasets (Datasets 1 and 2) were evaluated using the AUC metric with the eight methods (Table 1), their performance might not be generalizable in practice [79]. Indeed, a recent study reported the utility of MAS [82]. Also, a shared disadvantage of RMA and DFW is that the probeset intensities change when microarrays are re-preprocessed because of the inclusion of additional arrays, but modification strategies to deal with it have only been developed for RMA [81,88,89]. We therefore discuss the best methods for each preprocessing algorithm.

For MAS users, we think WAD is the most promising method because it gave good results for both types of dataset (artificial spike-in and real experimental datasets, see Tables 1, 2, and 4). The second best was ibmT [20]. Although there was no a statistically significant difference between the 36 AUC values for WAD from the real experimental datasets and those for the second best method (ibmT) (one-tail *p*-value = 0.18, paired *t*-test; see Table 7a), it is natural that one should select the best performing method for a number of real datasets.

**Table 7:** Statistical significance between two methods for Datasets 3–38

a) MAS		Inferior							
		
		WAD	AD	FC	RP	modT	samT	shrinkT	ibmT
Superior	WAD	-	**2.1E-07**	**6.7E-07**	**2.3E-06**	**2.2E-02**	**1.7E-02**	**2.0E-02**	1.8E-01
	AD	1.0E+00	-	8.1E-01	**2.9E-04**	1.0E+00	1.0E+00	1.0E+00	1.0E+00
	FC	1.0E+00	1.9E-01	-	**2.6E-04**	1.0E+00	1.0E+00	1.0E+00	1.0E+00
	RP	1.0E+00	1.0E+00	1.0E+00	-	1.0E+00	1.0E+00	1.0E+00	1.0E+00
	modT	9.8E-01	**8.6E-04**	**4.0E-03**	**1.4E-04**	-	4.7E-01	9.0E-01	1.0E+00
	samT	9.8E-01	**2.5E-04**	**2.0E-03**	**6.6E-05**	5.3E-01	-	6.9E-01	1.0E+00
	shrinkT	9.8E-01	**4.0E-04**	**2.2E-03**	**9.0E-05**	1.0E-01	3.1E-01	-	1.0E+00
	ibmT	8.2E-01	**4.7E-05**	**2.6E-04**	**2.6E-05**	**2.2E-04**	**2.3E-03**	**2.9E-04**	-

(b) RMA		Inferior							
		
		WAD	AD	FC	RP	modT	samT	shrinkT	ibmT

Superior	WAD	-	9.8E-01	9.8E-01	8.9E-01	3.0E-01	3.1E-01	2.5E-01	4.4E-01
	AD	**2.3E-02**	-	4.7E-01	8.3E-02	**9.2E-03**	**1.1E-02**	**7.2E-03**	**2.8E-02**
	FC	**2.4E-02**	5.3E-01	-	8.8E-02	**1.1E-02**	**1.3E-02**	**8.4E-03**	**3.1E-02**
	RP	1.1E-01	9.2E-01	9.1E-01	-	8.4E-02	9.7E-02	6.6E-02	1.7E-01
	modT	7.0E-01	9.9E-01	9.9E-01	9.2E-01	-	5.6E-01	6.5E-02	1.0E+00
	samT	6.9E-01	9.9E-01	9.9E-01	9.0E-01	4.4E-01	-	2.1E-01	8.3E-01
	shrinkT	7.5E-01	9.9E-01	9.9E-01	9.3E-01	9.4E-01	7.9E-01	-	1.0E+00
	ibmT	5.6E-01	9.7E-01	9.7E-01	8.3E-01	**3.2E-03**	1.7E-01	**1.9E-03**	-

(c) DFW		Inferior							
		
		WAD	AD	FC	RP	modT	samT	shrinkT	ibmT

Superior	WAD	-	1.0E+00	1.0E+00	1.0E+00	1.3E-01	1.2E-01	4.5E-01	1.6E-01
	AD	**2.5E-03**	-	1.6E-01	9.6E-01	5.1E-02	**4.7E-02**	2.1E-01	6.9E-02
	FC	**2.6E-03**	8.4E-01	-	9.6E-01	5.1E-02	**4.7E-02**	2.1E-01	6.9E-02
	RP	**8.7E-04**	**4.2E-02**	**4.1E-02**	-	**3.0E-02**	**3.0E-02**	1.1E-01	**4.4E-02**
	modT	8.7E-01	9.5E-01	9.5E-01	9.7E-01	-	8.6E-02	9.9E-01	8.5E-01
	samT	8.8E-01	9.5E-01	9.5E-01	9.7E-01	9.1E-01	-	9.9E-01	1.0E+00
	shrinkT	5.5E-01	7.9E-01	7.9E-01	8.9E-01	**6.1E-03**	**1.0E-02**	-	**2.6E-02**
	ibmT	8.4E-01	9.3E-01	9.3E-01	9.6E-01	1.5E-01	**5.2E-04**	9.7E-01	-

The *p*-values between the 36 AUC values from a possibly superior method and those from a possibly inferior method were calculated by a one-tail paired *t*-test. The null hypothesis is that the mean of the 36 AUC values for one method is the same as that for the other method. There are two *p*-values for two methods compared. For example, in (a) MAS-preprocessed data, the *p*-value is 1.8E-01 when the alternative hypothesis is that the mean of the 36 AUC values for WAD is greater than that for ibmT while the *p*-value is 8.2E-01 when the alternative hypothesis is that the mean of the 36 AUC values for ibmT is greater than that for WAD. Combinations having *p *< 0.05 are highlighted in bold.

For RMA users, FC-based methods can be recommended. Although these methods (except WAD) were inferior to the *t*-statistic-based methods when the results for the older spike-in dataset (Dataset 1, which is obtained from the HG-U95A array) were compared, they were better for both the newer spike-in dataset (Dataset 2, which is from the HG-U133A array) and the 36 real experimental datasets (Datasets 3–38, which is also from the HG-U133A array). We think that the results for the real experimental datasets (or a newer platform) should take precedence over the results for the artificial datasets (or an older platform). AD or FC may be the best since they are the best for the 36 real datasets (see Tables 4 and 7b).

For DFW users, RP can be recommended since it was the best for the 36 real experimental datasets (see Tables 4 and 7c). However, the use of RP for analyzing large numbers of arrays can be sometimes limited by available computer memory. The other FC-based methods can be recommended for such a situation.

The variance estimation is much more challenging when the number of replicates is small [29]. This suggests that the FC-based methods including WAD tend to be more powerful (or less powerful) than the *t*-statistic-based methods if the number of replicates is small (or large). We found that WAD was the best for some datasets which contain large (> 10) replicates (*e.g*., Datasets 5, 7, and 26) while FC and RP tended to perform the best on datasets with relatively small replicates (*e.g*., Datasets 34 and 10, whose numbers of replicates in one class were smaller than 6) [see Additional file 1]. These results suggest that WAD can perform well across a range of replicate numbers.

It is important to mention that there are other preprocessing algorithms such as FARMS [39] and SuperNorm [90]. FARMS considers data on a multi-array basis as does RMA and DFW, while SuperNorm considers data on a per-array basis as does MAS. Although the FC-based methods were superior to the *t*-statistic-based methods, the latter methods might perform well for FARMS- or SuperNorm-preprocessed data. The evaluation of competing methods for these preprocessing algorithms will be our next task.

In practice, one may want to detect the DEGs from gene expression data, produced from a comparison of two or more classes (or time points), and the current method does not analyze these DEGs. A simple way to deal with them is to use $AD(i)=\max(\bar{x_{i}^{q}})-\min(\bar{x_{i}^{q}})$ and $\bar{x_{i}}=mean(\bar{x_{i}^{q}})$ in WAD for the *q *class problem (*q *= 1, 2, 3, ...) (see the Methods section for details). Of course, there are many possible ways to analyze these DEGs. Further work is needed to make WAD universal.

## Conclusion

We proposed a new method (called WAD) for ranking differentially expressed genes (DEGs) from gene expression data, especially obtained by Affymetrix GeneChip^® ^technology. The basic assumption for WAD was that strong signals are better signals. We demonstrated that known or potential marker genes had high expression levels on average in 34 of the 36 real experimental datasets and applied our idea as the weight term in the WAD statistic.

Overall, WAD was more powerful than the other methods in terms of the area under the receiver operating characteristic curve. WAD also gave consistent results for different preprocessing algorithms. Its performance was verified using a total of 38 artificial spike-in datasets and real experimental datasets. Given its excellent performance, we believe that WAD should become one of the methods used for analyzing microarray data.

## Methods

### Microarray data

The processed data (MAS-, RMA-, and DFW-preprocessed data) for Datasets 1 and 2 were downloaded from the Affycomp II website [42]. The raw (probe level) data for Dataset 3–38 were obtained from the Gene Expression Omnibus (GEO) website [78]. All analyses were performed using log_2_-transformed data except for the FC analysis. In Datasets 3–38, the 'true' DEGs were defined as those differential expressions that had been confirmed by real-time polymerase chain reaction (RT-PCR). For example, we defined 16 probesets (corresponding to 15 genes) of 20 candidates as DEGs in Dataset 9 [48] because the remaining four probesets (or genes) showed incompatible expression patterns between RT-PCR and the microarray. For reproducibility, detailed information on these datasets is given in the additional file [see Additional file 1].

### Weighted Average Difference (WAD) method

Consider a gene expression matrix consisting of *p *genes and *n *arrays, produced from a comparison between classes A and B. The average difference ($AD_{i}=\bar{x_{i}^{B}}-\bar{x_{i}^{A}}$), defined here as the average log signal for all class B replicates ($\bar{x_{i}^{B}}$) minus the average log signal for all class A replicates ($\bar{x_{i}^{A}}$), is an obvious indicator for estimating the differential expression of the *i*th gene, $x_{i}=(x_{i}^{1},...,x_{i}^{n})$. Some of the top-ranked genes from the simple statistic, however, tend to exhibit lower expression levels. This is not good because the signal-to-noise ratio decreases with the gene expression level [3] and because known DEGs tend to have high expression levels.

To account for these observations, we use relative average log signal intensity *w*_*i *_for weighting the average difference in ***x***_*i*_.

(1) $$w_{i}=\frac{\bar{x_{i}}-min}{max-min},$$

where $\bar{x_{i}}$ is calculated as $(\bar{x_{i}^{A}}+\bar{x_{i}^{B}})/2$, and the *max *(or *min*) indicates the maximum (or minimum) value in an average expression vector $\left(\bar{x_{1}},...,\bar{x_{p}}\right)$ on a log scale.

The WAD statistic for the *i*th gene, *WAD*(*i*), is calculated simply as

(2) *WAD*(*i*) = *AD*_*i *_× *w*_*i*_.

The basic assumption for our approach to the gene ranking problem is that ''strong signals are better signals'' [36]. The WAD statistic is a straightforward application of this idea. The R-source codes for analyzing Datasets 1 and 2 are available in additional files [see Additional files 2 and 3].

### Fold change (FC) method

The FC statistic for the *i*th gene, *FC*(*i*), was calculated as the average non-log signal for all class B replicates divided by the average non-log signal for all class A replicates. The ranking for selecting DEGs was performed using the log of *FC*(*i*).

### Rank products (RP) method

The RP method is an FC-based method. The RP statistic was calculated using the RP() function in the "RankProd" library [37] in R [75] and Bioconductor [76].

### Moderated *t*-statistic (modT) method

The modT method is an empirical Bayes modification of the *t*-test [9]. The modT statistic was calculated using the modt.stat() function in the "st" library [23] in R [75].

### Significance analysis of microarrays (samT) method

The samT method is a modification of the *t*-test [3], and it works by adding a small value to the denominator of the *t *statistic. The samT statistic was calculated using the sam.stat() function in the "st" library [23] in R [75].

### Shrinkage *t*-statistic (shrinkT) method

The shrinkT method is a quasi-empirical Bayes modification of the *t*-test [23]. The shrinkT statistic was calculated using the shrinkt.stat() function in the "st" library [23] in R [75].

### Intensity-based moderated *t*-statistic (ibmT) method

The ibmT method is a modified version of the modT method [20]. The ibmT statistic was calculated using the IBMT() function, available on-line [91].

## Abbreviations

AUC: area under ROC curve; DEG: differentially expressed gene; DFW: distribution-free weighted (method); FC: fold change; FP: false positive; ibmT: intensity-based moderated *t*-statistic; MAS: (Affymetrix) MicroArray Suite version 5; modT: moderated *t*-statistic; RMA: robust multi-chip average; ROC: receiver operating characteristic; RP: rank products; samT: significance analysis of microarrays; shrinkT: shrinkage *t*-statistic; TP: true positive; WAD: weighted average difference (method)

## Authors' contributions

KK developed the method and wrote the paper, YN and KS provided critical comments and led the project.

## Supplementary Material

Additional file 1 Detailed information for Datasets 3–38. Click here for file

Additional file 2 R-code for analyzing Dataset 1. Click here for file

Additional file 3 R-code for analyzing Dataset 2. Click here for file

Additional file 4 Average expression vectors and the results of outlier detection for Datasets 3–26. Sheet 1: Average expression vectors are provided. Sheet 2: For each of the original average expression vectors, an outlier vector (consisting of 1 for over-expressed outliers, -1 for under-expressed outliers, and 0 for non-outliers) is provided. This sheet does not contain "-1". Click here for file

Additional file 5 Average expression vectors and the results of outlier detection for Datasets 27–38. Sheet 1: Average expression vectors are provided. Sheet 2: For each of the original average expression vectors, an outlier vector (consisting of 1 for over-expressed outliers, -1 for under-expressed outliers, and 0 for non-outliers) is provided. This sheet does not contain "-1". Click here for file
