# Supplementary material for: A weighted average difference method for detecting differentially expressed genes from microarray data
Source: Algorithms Mol Biol. 2008 Jun 26;3:8. doi: 10.1186/1748-7188-3-8 (PMC2464587; doi:10.1186/1748-7188-3-8)
Supplement: Additional file 1 — Detailed information for Datasets 3–38. [file 1748-7188-3-8-S1.doc]

**Detailed information for Datasets 3-38**

**Dataset serial number**

**1. PubMed ID for the original paper:**

**2. Gene Expression Omnibus (GEO) series ID:**

**3. GEO sample IDs in one state:**

**4. GEO sample IDs in the other state:**

**5. Number of probesets and the IDs confirmed by RT-PCR:**

**6. Preprocessing algorithm used in the original study:**

**7. Preprocessing in this study:**

**8. Results**

**8-1. Percentile ranks for true positives**

**8-2. AUC values**

**Dataset 3**

**1.** http://www.ncbi.nlm.nih.gov/sites/entrez?Db=Pubmed&term=15728662[UID]

**2.** GSE1462

**3.** 3 normal subjects:

GSM24652-24654

**4.** 4 mtDNA “Common”-deletion subjects:

GSM24663-24666

**5.** 4 probesets.

"204388_s_at", "204173_at", "204570_at", "212160_at"

**6.** MAS5.0

**7.** RMA- and DFW-preprocessed data were obtained from a total of **15** samples in GSE1462.

**8-1.**

| MAS-preprocessed data | | |  |  |  |
| --- | --- | --- | --- | --- | --- |
| Method | 0% | 25% | 50% | 75% | 100% |
| *w* | 52.00 | 222.25 | 3770.50 | 7926.25 | 9919.00 |
| AD | 37.00 | 370.00 | 514.00 | 2537.00 | 8507.00 |
| WAD | 9.00 | 12.00 | 45.50 | 560.75 | 2009.00 |
| FC | 171.00 | 434.25 | 727.50 | 2869.50 | 8679.00 |
| RP | 15.00 | 411.00 | 583.50 | 3190.25 | 10889.00 |
| modT | 117.00 | 507.75 | 1740.50 | 3276.50 | 4577.00 |
| samT | 137.00 | 584.75 | 1706.00 | 3065.75 | 4229.00 |
| shrinkT | 121.00 | 565.75 | 1949.00 | 3428.00 | 4160.00 |
| ibmT | 24.00 | 136.50 | 928.00 | 1816.25 | 2219.00 |
| RMA-preprocessed data | | |  |  |  |
| Method | 0% | 25% | 50% | 75% | 100% |
| *w* | 59.00 | 168.50 | 2611.00 | 7173.50 | 13643.00 |
| AD | 11.00 | 20.00 | 23.50 | 3745.50 | 14910.00 |
| WAD | 4.00 | 11.50 | 48.00 | 1627.25 | 6263.00 |
| FC | 17.00 | 24.50 | 38.50 | 3815.50 | 15112.00 |
| RP | 14.00 | 15.50 | 19.50 | 4677.00 | 18639.00 |
| modT | 22.00 | 405.25 | 1968.00 | 5563.50 | 12045.00 |
| samT | 18.00 | 246.75 | 1281.00 | 4573.50 | 11577.00 |
| shrinkT | 76.00 | 780.25 | 2866.50 | 6231.50 | 10772.00 |
| ibmT | 13.00 | 316.75 | 1693.00 | 5273.00 | 12188.00 |
| DFW-preprocessed data | | |  |  |  |
| Method | 0% | 25% | 50% | 75% | 100% |
| *w* | 72.00 | 447.75 | 7769.50 | 15132.75 | 15633.00 |
| AD | 11.00 | 29.75 | 126.00 | 2345.75 | 8735.00 |
| WAD | 4.00 | 51.25 | 252.00 | 1566.50 | 4955.00 |
| FC | 11.00 | 29.00 | 126.00 | 2346.75 | 8736.00 |
| RP | 9.00 | 56.25 | 77.00 | 2303.00 | 8966.00 |
| modT | 303.00 | 1588.50 | 3779.00 | 6843.75 | 10752.00 |
| samT | 426.00 | 1884.75 | 4219.50 | 7393.75 | 11371.00 |
| shrinkT | 126.00 | 795.00 | 2178.00 | 4608.00 | 8418.00 |
| ibmT | 297.00 | 1560.75 | 3687.50 | 6821.50 | 11107.00 |

**8-2.**

| Method | MAS | RMA | DFW |
| --- | --- | --- | --- |
| *w* | 0.80360 | 0.78776 | 0.64951 |
| AD | 0.89270 | 0.83215 | 0.89914 |
| WAD | 0.97645 | 0.92871 | 0.93881 |
| FC | 0.88448 | 0.82948 | 0.89913 |
| RP | 0.86466 | 0.79036 | 0.89767 |
| modT | 0.90838 | 0.82054 | 0.79125 |
| samT | 0.91283 | 0.84125 | 0.77304 |
| shrinkT | 0.90833 | 0.81405 | 0.85536 |
| ibmT | 0.95412 | 0.82521 | 0.78939 |

**Dataset 4**

**1.** http://www.ncbi.nlm.nih.gov/sites/entrez?Db=Pubmed&term=17555786[UID]

**2.** GSE7819

**3.** 3 SVG-A:

GSM189708-189710

**4.** 3 SVGR2:

GSM189711-189713

**5.** 11 probesets.

"212154_at", "212157_at", "212158_at", "205207_at", "209239_at",

"208200_at", "210118_s_at", "205798_at", "210073_at", "202859_x_at",

"211506_s_at"

**6.** MAS5.0

**7.** RMA- and DFW-preprocessed data were obtained from a total of 6 samples in GSE7819.

**8-1.**

| MAS-preprocessed data | | |  |  |  |
| --- | --- | --- | --- | --- | --- |
| Method | 0% | 25% | 50% | 75% | 100% |
| *w* | 1722.00 | 3577.00 | 4765.00 | 12466.50 | 19550.00 |
| AD | 1.00 | 41.50 | 398.00 | 1625.50 | 3572.00 |
| WAD | 1.00 | 20.50 | 184.00 | 278.50 | 2254.00 |
| FC | 1.00 | 76.50 | 413.00 | 1668.50 | 3409.00 |
| RP | 1.00 | 41.00 | 370.00 | 1702.50 | 3179.00 |
| modT | 8.00 | 131.00 | 343.00 | 539.00 | 879.00 |
| samT | 4.00 | 101.50 | 267.00 | 558.00 | 662.00 |
| shrinkT | 10.00 | 132.50 | 400.00 | 529.50 | 1025.00 |
| ibmT | 3.00 | 44.00 | 249.00 | 418.50 | 2919.00 |
| RMA-preprocessed data | | |  |  |  |
| Method | 0% | 25% | 50% | 75% | 100% |
| *w* | 1825.00 | 3746.50 | 5231.00 | 14517.50 | 21401.00 |
| AD | 1.00 | 21.50 | 152.00 | 1023.50 | 14381.00 |
| WAD | 2.00 | 10.50 | 67.00 | 2158.50 | 16105.00 |
| FC | 1.00 | 22.50 | 176.00 | 931.00 | 14193.00 |
| RP | 1.00 | 27.50 | 113.00 | 1230.00 | 14071.00 |
| modT | 7.00 | 79.00 | 370.00 | 1584.50 | 14668.00 |
| samT | 17.00 | 168.50 | 617.00 | 2260.50 | 15144.00 |
| shrinkT | 16.00 | 174.00 | 592.00 | 2136.50 | 14904.00 |
| ibmT | 5.00 | 70.50 | 340.00 | 1503.50 | 14542.00 |
| DFW-preprocessed data | | |  |  |  |
| Method | 0% | 25% | 50% | 75% | 100% |
| *w* | 2642.00 | 5261.00 | 11520.00 | 16620.00 | 21931.00 |
| AD | 2.00 | 16.00 | 75.00 | 1875.00 | 20807.00 |
| WAD | 2.00 | 21.00 | 56.00 | 2253.50 | 21015.00 |
| FC | 2.00 | 16.00 | 75.00 | 1875.00 | 20807.00 |
| RP | 3.00 | 21.50 | 44.00 | 1762.50 | 20621.00 |
| modT | 52.00 | 491.00 | 1100.00 | 1435.50 | 20030.00 |
| samT | 72.00 | 573.50 | 1241.00 | 1632.00 | 20101.00 |
| shrinkT | 44.00 | 413.00 | 942.00 | 1242.00 | 20490.00 |
| ibmT | 53.00 | 476.50 | 1071.00 | 1409.50 | 19671.00 |

**8-2.**

| Method | MAS | RMA | DFW |
| --- | --- | --- | --- |
| *w* | 0.63947 | 0.60475 | 0.49218 |
| AD | 0.95504 | 0.91651 | 0.86567 |
| WAD | 0.98193 | 0.85431 | 0.84263 |
| FC | 0.95497 | 0.91815 | 0.86568 |
| RP | 0.95659 | 0.91629 | 0.87369 |
| modT | 0.98486 | 0.90729 | 0.87569 |
| samT | 0.98618 | 0.89320 | 0.86958 |
| shrinkT | 0.98380 | 0.89663 | 0.87730 |
| ibmT | 0.97893 | 0.91262 | 0.87825 |

**Dataset 5**

**1.** http://www.ncbi.nlm.nih.gov/sites/entrez?Db=Pubmed&term=17490972[UID]

**2.** GSE8441

**3.** 11 control groups:

GSM209811, GSM209813, GSM209815, GSM209817, GSM209819,

GSM209821, GSM209823, GSM209825, GSM209827, GSM209829,

GSM209831

**4.** 11 inadequate dietary protein intake groups:

GSM209812, GSM209814, GSM209816, GSM209818, GSM209820,

GSM209822, GSM209824, GSM209826, GSM209828, GSM209830,

GSM209832

**5.** 9 probesets.

"201721_s_at", "221232_s_at", "211025_x_at", "211699_x_at", "204802_at",

"208581_x_at", "202310_s_at", "204051_s_at", "215049_x_at"

**6.** MAS5.0

**7.** RMA- and DFW-preprocessed data were obtained from a total of 22 samples in GSE8441.

**8-1.**

| MAS-preprocessed data | | |  |  |  |
| --- | --- | --- | --- | --- | --- |
| Method | 0% | 25% | 50% | 75% | 100% |
| *w* | 221.00 | 625.00 | 1599.00 | 5336.00 | 7899.00 |
| AD | 8.00 | 1010.00 | 1533.00 | 2734.00 | 4888.00 |
| WAD | 1.00 | 26.00 | 63.00 | 220.00 | 721.00 |
| FC | 2.00 | 260.00 | 1307.00 | 1701.00 | 9632.00 |
| RP | 21.00 | 1190.00 | 3193.00 | 5248.00 | 7303.00 |
| modT | 12.00 | 70.00 | 191.00 | 1827.00 | 7349.00 |
| samT | 12.00 | 77.00 | 224.00 | 1722.00 | 7234.00 |
| shrinkT | 12.00 | 88.00 | 373.00 | 1462.00 | 6612.00 |
| ibmT | 7.00 | 33.00 | 70.00 | 1237.00 | 6293.00 |
| RMA-preprocessed data | | |  |  |  |
| Method | 0% | 25% | 50% | 75% | 100% |
| *w* | 128.00 | 520.00 | 2020.00 | 7572.00 | 9962.00 |
| AD | 1.00 | 23.00 | 51.00 | 68.00 | 144.00 |
| WAD | 1.00 | 18.00 | 44.00 | 133.00 | 444.00 |
| FC | 1.00 | 27.00 | 64.00 | 74.00 | 532.00 |
| RP | 1.00 | 22.00 | 37.00 | 172.00 | 202.00 |
| modT | 7.00 | 63.00 | 189.00 | 1846.00 | 6753.00 |
| samT | 1.00 | 35.00 | 108.00 | 1301.00 | 5454.00 |
| shrinkT | 14.00 | 76.00 | 214.00 | 1965.00 | 6996.00 |
| ibmT | 3.00 | 48.00 | 135.00 | 1568.00 | 6409.00 |
| DFW-preprocessed data | | |  |  |  |
| Method | 0% | 25% | 50% | 75% | 100% |
| *w* | 263.00 | 865.00 | 2046.00 | 5229.00 | 16648.00 |
| AD | 1.00 | 17.00 | 52.00 | 359.00 | 805.00 |
| WAD | 1.00 | 17.00 | 41.00 | 659.00 | 1052.00 |
| FC | 1.00 | 17.00 | 52.00 | 359.00 | 804.00 |
| RP | 1.00 | 16.00 | 54.00 | 118.00 | 387.00 |
| modT | 67.00 | 92.00 | 203.00 | 2615.00 | 8836.00 |
| samT | 60.00 | 84.00 | 180.00 | 2463.00 | 8619.00 |
| shrinkT | 33.00 | 60.00 | 129.00 | 1750.00 | 6918.00 |
| ibmT | 58.00 | 81.00 | 182.00 | 2519.00 | 8704.00 |

**8-2.**

| Method | MAS | RMA | DFW |
| --- | --- | --- | --- |
| *w* | 0.87696 | 0.83945 | 0.78165 |
| AD | 0.91122 | 0.99783 | 0.99129 |
| WAD | 0.99102 | 0.99555 | 0.98709 |
| FC | 0.90012 | 0.99497 | 0.99129 |
| RP | 0.84457 | 0.99664 | 0.99506 |
| modT | 0.93756 | 0.93206 | 0.91049 |
| samT | 0.93863 | 0.94945 | 0.91428 |
| shrinkT | 0.94407 | 0.92842 | 0.93496 |
| ibmT | 0.95147 | 0.93856 | 0.91299 |

**Dataset 6**

**1.** http://www.ncbi.nlm.nih.gov/sites/entrez?Db=Pubmed&term=18029387[UID]

**2.** GSE9499

**3.** 15 normal cases: GSM241000-GSM241014

**4.** 7 ICF syndrome cases: GSM241015-GSM241021

**5.**  77 probesets.

"215356_at", "213194_at", "204201_s_at", "205403_at", "211372_s_at",

"201122_x_at", "201123_s_at", "204866_at", "207426_s_at", "204072_s_at",

"209582_s_at", "209583_s_at", "201565_s_at", "206337_at", "206140_at",

"211219_s_at", "206571_s_at", "218181_s_at", "204897_at", "204689_at",

"215933_s_at", "205991_s_at", "212464_s_at", "211719_x_at", "216442_x_at",

"210495_x_at", "177_at", "215723_s_at", "201876_at", "210830_s_at",

"218229_s_at", "220643_s_at", "206489_s_at", "206490_at", "205039_s_at",

"221773_at", "221210_s_at", "202674_s_at", "220188_at", "213103_at",

"210629_x_at", "215633_x_at", "214574_x_at", "205290_s_at", "201349_at",

"206150_at", "204445_s_at", "204446_s_at", "214366_s_at", "206513_at",

"209771_x_at", "216379_x_at", "266_s_at", "205124_at", "213236_at",

"41644_at", "209079_x_at", "205717_x_at", "211066_x_at", "205098_at",

"205099_s_at", "204890_s_at", "204891_s_at", "209569_x_at", "211577_s_at",

"33304_at", "204698_at", "217983_s_at", "217984_at", "209269_s_at",

"207540_s_at", "205484_at", "213664_at", "204159_at", "210992_x_at",

"211395_x_at", "211644_x_at"

**6.** MAS5.0

**7.** RMA- and DFW-preprocessed data were obtained from a total of 22 samples in GSE9499.

**8-1.**

| MAS-preprocessed data | | |  |  |  |
| --- | --- | --- | --- | --- | --- |
| Method | 0% | 25% | 50% | 75% | 100% |
| *w* | 588.00 | 6798.00 | 9214.00 | 12706.00 | 18094.00 |
| AD | 4.00 | 60.00 | 220.00 | 537.00 | 8822.00 |
| WAD | 4.00 | 72.00 | 150.00 | 301.00 | 11356.00 |
| FC | 8.00 | 66.00 | 255.00 | 597.00 | 15188.00 |
| RP | 3.00 | 56.00 | 263.00 | 556.00 | 8106.00 |
| modT | 2.00 | 34.00 | 103.00 | 276.00 | 14294.00 |
| samT | 2.00 | 38.00 | 86.00 | 203.00 | 13328.00 |
| shrinkT | 2.00 | 34.00 | 91.00 | 236.00 | 14048.00 |
| ibmT | 2.00 | 35.00 | 96.00 | 300.00 | 14374.00 |
| RMA-preprocessed data | | |  |  |  |
| Method | 0% | 25% | 50% | 75% | 100% |
| *w* | 656.00 | 5654.00 | 10062.00 | 14064.00 | 20683.00 |
| AD | 3.00 | 69.00 | 154.00 | 318.00 | 19759.00 |
| WAD | 6.00 | 65.00 | 348.00 | 934.00 | 20658.00 |
| FC | 5.00 | 74.00 | 163.00 | 316.00 | 19075.00 |
| RP | 3.00 | 42.00 | 124.00 | 337.00 | 16757.00 |
| modT | 1.00 | 46.00 | 99.00 | 192.00 | 18826.00 |
| samT | 1.00 | 41.00 | 83.00 | 175.00 | 18949.00 |
| shrinkT | 1.00 | 45.00 | 103.00 | 204.00 | 18762.00 |
| ibmT | 1.00 | 45.00 | 91.00 | 180.00 | 18638.00 |
| DFW-preprocessed data | | |  |  |  |
| Method | 0% | 25% | 50% | 75% | 100% |
| *w* | 1228.00 | 6999.00 | 13018.00 | 16684.00 | 21771.00 |
| AD | 8.00 | 184.00 | 829.00 | 1916.00 | 21740.00 |
| WAD | 15.00 | 247.00 | 1454.00 | 2700.00 | 21368.00 |
| FC | 6.00 | 184.00 | 829.00 | 1915.00 | 21740.00 |
| RP | 3.00 | 94.00 | 455.00 | 1411.00 | 18311.00 |
| modT | 1.00 | 34.00 | 149.00 | 280.00 | 21870.00 |
| samT | 1.00 | 34.00 | 147.00 | 273.00 | 21880.00 |
| shrinkT | 1.00 | 33.00 | 140.00 | 265.00 | 21786.00 |
| ibmT | 1.00 | 33.00 | 147.00 | 276.00 | 21865.00 |

**8-2.**

| Method | MAS | RMA | DFW |
| --- | --- | --- | --- |
| *w* | 0.58720 | 0.54632 | 0.45435 |
| AD | 0.97683 | 0.96773 | 0.92229 |
| WAD | 0.98345 | 0.95064 | 0.90218 |
| FC | 0.97178 | 0.96750 | 0.92229 |
| RP | 0.97305 | 0.97217 | 0.94333 |
| modT | 0.98137 | 0.97267 | 0.96776 |
| samT | 0.98465 | 0.97360 | 0.96795 |
| shrinkT | 0.98255 | 0.97241 | 0.96820 |
| ibmT | 0.98140 | 0.97324 | 0.96800 |

**Dataset 7**

**1.** http://www.ncbi.nlm.nih.gov/sites/entrez?Db=Pubmed&term=14872006[UID]

**2.** GSE974

**3.** 19 pre:

GSM14844, GSM14938, GSM14940, GSM14942, GSM14944,

GSM14946, GSM14948, GSM14950, GSM14952, GSM14954,

GSM14956, GSM14958, GSM14960, GSM14962, GSM14965,

GSM14967, GSM14969, GSM14971, GSM14973

**4.** 19 post:

GSM14936, GSM14937, GSM14939, GSM14941, GSM14943,

GSM14945, GSM14947, GSM14949, GSM14951, GSM14953,

GSM14955, GSM14957, GSM14959, GSM14961, GSM14963,

GSM14966, GSM14968, GSM14970, GSM14972

**5.** 3 probesets.

"212558_at", "212298_at", "205517_at"

**6.** MAS5.0

**7.** RMA- and DFW-preprocessed data were obtained from a total of 38 samples in GSE974.

**8-1.**

| MAS-preprocessed data | | |  |  |  |
| --- | --- | --- | --- | --- | --- |
| Method | 0% | 25% | 50% | 75% | 100% |
| *w* | 2308.00 | 2723.50 | 3139.00 | 3810.50 | 4482.00 |
| AD | 1266.00 | 1394.00 | 1522.00 | 2827.50 | 4133.00 |
| WAD | 382.00 | 391.00 | 400.00 | 1143.50 | 1887.00 |
| FC | 1561.00 | 1975.50 | 2390.00 | 2404.00 | 2418.00 |
| RP | 3461.00 | 4286.00 | 5111.00 | 5161.00 | 5211.00 |
| modT | 54.00 | 109.50 | 165.00 | 1473.00 | 2781.00 |
| samT | 87.00 | 151.50 | 216.00 | 1516.00 | 2816.00 |
| shrinkT | 80.00 | 137.00 | 194.00 | 1451.00 | 2708.00 |
| ibmT | 46.00 | 94.00 | 142.00 | 1322.00 | 2502.00 |
| RMA-preprocessed data | | |  |  |  |
| Method | 0% | 25% | 50% | 75% | 100% |
| *w* | 1703.00 | 2845.50 | 3988.00 | 5094.00 | 6200.00 |
| AD | 82.00 | 301.50 | 521.00 | 901.00 | 1281.00 |
| WAD | 150.00 | 251.00 | 352.00 | 892.00 | 1432.00 |
| FC | 213.00 | 565.00 | 917.00 | 1012.00 | 1107.00 |
| RP | 141.00 | 315.50 | 490.00 | 615.50 | 741.00 |
| modT | 9.00 | 221.50 | 434.00 | 1846.00 | 3258.00 |
| samT | 10.00 | 206.50 | 403.00 | 1694.00 | 2985.00 |
| shrinkT | 10.00 | 192.00 | 374.00 | 1614.50 | 2855.00 |
| ibmT | 10.00 | 234.00 | 458.00 | 1837.00 | 3216.00 |
| DFW-preprocessed data | | |  |  |  |
| Method | 0% | 25% | 50% | 75% | 100% |
| *w* | 3337.00 | 4224.50 | 5112.00 | 11061.50 | 17011.00 |
| AD | 665.00 | 823.00 | 981.00 | 1051.50 | 1122.00 |
| WAD | 663.00 | 869.50 | 1076.00 | 1304.50 | 1533.00 |
| FC | 661.00 | 818.50 | 976.00 | 1045.50 | 1115.00 |
| RP | 372.00 | 540.00 | 708.00 | 779.00 | 850.00 |
| modT | 7.00 | 150.50 | 294.00 | 831.00 | 1368.00 |
| samT | 6.00 | 143.50 | 281.00 | 798.50 | 1316.00 |
| shrinkT | 5.00 | 118.00 | 231.00 | 638.50 | 1046.00 |
| ibmT | 7.00 | 147.00 | 287.00 | 817.00 | 1347.00 |

**8-2.**

| Method | MAS | RMA | DFW |
| --- | --- | --- | --- |
| *w* | 0.85154 | 0.82219 | 0.61918 |
| AD | 0.89654 | 0.97190 | 0.95868 |
| WAD | 0.96016 | 0.97115 | 0.95114 |
| FC | 0.90480 | 0.96662 | 0.95892 |
| RP | 0.79388 | 0.97956 | 0.97121 |
| modT | 0.95521 | 0.94472 | 0.97512 |
| samT | 0.95343 | 0.94925 | 0.97611 |
| shrinkT | 0.95548 | 0.95163 | 0.98091 |
| ibmT | 0.95984 | 0.94497 | 0.97554 |

**Dataset 8**

**1.** http://www.ncbi.nlm.nih.gov/sites/entrez?Db=Pubmed&term=16617158[UID]

**2.** GSE2638 and GSE2639

**3.** 7 control samples:

GSM50771-50773 and GSM50777-50780

**4.** 7 TNF-stimulated samples:

GSM50774-50776 and GSM50781-50784

**5.** 13 probesets.

"211506_s_at", "216598_s_at", "214974_x_at", "204470_at", "210511_s_at",

"205992_s_at", "209716_at", "207850_at", "205476_at", "205798_at",

"207196_s_at", "212641_at", "212642_s_at"

**6.** MAS5.0

**7.** RMA- and DFW-preprocessed data were obtained from a total of 14 samples in GSE2638 and GSE2639.

**8-1.**

| MAS-preprocessed data | | |  |  |  |
| --- | --- | --- | --- | --- | --- |
| Method | 0% | 25% | 50% | 75% | 100% |
| *w* | 429.00 | 2208.00 | 6561.00 | 9797.00 | 13141.00 |
| AD | 1.00 | 18.00 | 40.00 | 63.00 | 113.00 |
| WAD | 2.00 | 15.00 | 23.00 | 52.00 | 89.00 |
| FC | 1.00 | 25.00 | 51.00 | 99.00 | 161.00 |
| RP | 1.00 | 19.00 | 45.00 | 74.00 | 162.00 |
| modT | 1.00 | 15.00 | 32.00 | 56.00 | 137.00 |
| samT | 1.00 | 15.00 | 28.00 | 49.00 | 103.00 |
| shrinkT | 1.00 | 15.00 | 31.00 | 54.00 | 133.00 |
| ibmT | 1.00 | 13.00 | 33.00 | 56.00 | 154.00 |
| RMA-preprocessed data | | |  |  |  |
| Method | 0% | 25% | 50% | 75% | 100% |
| *w* | 380.00 | 2643.00 | 6914.00 | 9896.00 | 15293.00 |
| AD | 3.00 | 12.00 | 25.00 | 81.00 | 160.00 |
| WAD | 3.00 | 16.00 | 40.00 | 96.00 | 495.00 |
| FC | 3.00 | 15.00 | 29.00 | 116.00 | 205.00 |
| RP | 2.00 | 12.00 | 21.00 | 79.00 | 150.00 |
| modT | 1.00 | 6.00 | 29.00 | 57.00 | 102.00 |
| samT | 1.00 | 8.00 | 33.00 | 50.00 | 84.00 |
| shrinkT | 1.00 | 6.00 | 31.00 | 65.00 | 118.00 |
| ibmT | 1.00 | 6.00 | 31.00 | 51.00 | 92.00 |
| DFW-preprocessed data | | |  |  |  |
| Method | 0% | 25% | 50% | 75% | 100% |
| *w* | 1216.00 | 5689.00 | 8508.00 | 15687.00 | 19228.00 |
| AD | 4.00 | 26.00 | 57.00 | 1069.00 | 1681.00 |
| WAD | 3.00 | 18.00 | 86.00 | 1449.00 | 2009.00 |
| FC | 4.00 | 27.00 | 59.00 | 1066.00 | 1682.00 |
| RP | 3.00 | 19.00 | 30.00 | 469.00 | 702.00 |
| modT | 2.00 | 8.00 | 27.00 | 68.00 | 144.00 |
| samT | 2.00 | 8.00 | 27.00 | 71.00 | 145.00 |
| shrinkT | 2.00 | 8.00 | 27.00 | 68.00 | 142.00 |
| ibmT | 2.00 | 8.00 | 27.00 | 69.00 | 141.00 |

**8-2.**

| Method | MAS | RMA | DFW |
| --- | --- | --- | --- |
| *w* | 0.69648 | 0.67361 | 0.55169 |
| AD | 0.99833 | 0.99798 | 0.97902 |
| WAD | 0.99878 | 0.99592 | 0.97386 |
| FC | 0.99760 | 0.99728 | 0.97897 |
| RP | 0.99783 | 0.99809 | 0.99105 |
| modT | 0.99861 | 0.99861 | 0.99801 |
| samT | 0.99883 | 0.99889 | 0.99798 |
| shrinkT | 0.99864 | 0.99847 | 0.99804 |
| ibmT | 0.99851 | 0.99874 | 0.99803 |

**Dataset 9**

**1.** http://www.ncbi.nlm.nih.gov/sites/entrez?Db=Pubmed&term=16617158[UID]

**2.** GSE2638 and GSE2639

**3.** 3 HMEC TNF-stimulated samples:

GSM50774-50776

**4.** 4 HUVEC TNF-stimulated samples:

GSM50781-50784

**5.** 16 probesets.

"205289_at", "206336_at", "209774_x_at", "203687_at", "210228_at",

"210229_s_at", "206618_at", "202688_at", "203917_at", "204748_at",

"221085_at", "221009_s_at", "204475_at", "211959_at", "206026_s_at",

"204580_at"

**6.** MAS5.0

**7.** RMA- and DFW-preprocessed data were obtained from a total of **14** samples in GSE2638 and GSE2639.

**8-1.**

| MAS-preprocessed data | | |  |  |  |
| --- | --- | --- | --- | --- | --- |
| Method | 0% | 25% | 50% | 75% | 100% |
| *w* | 2540.00 | 7564.50 | 8893.50 | 11553.75 | 15354.00 |
| AD | 5.00 | 40.25 | 2657.00 | 7016.00 | 13319.00 |
| WAD | 10.00 | 78.00 | 996.00 | 4759.75 | 12278.00 |
| FC | 5.00 | 51.75 | 2211.50 | 7191.25 | 14414.00 |
| RP | 9.00 | 51.75 | 3273.50 | 4627.75 | 14717.00 |
| modT | 12.00 | 554.75 | 1705.50 | 5083.25 | 15036.00 |
| samT | 6.00 | 131.00 | 1719.50 | 5545.25 | 13317.00 |
| shrinkT | 9.00 | 470.00 | 1576.00 | 5076.50 | 14777.00 |
| ibmT | 13.00 | 539.00 | 1180.00 | 4380.00 | 15437.00 |
| RMA-preprocessed data | | |  |  |  |
| Method | 0% | 25% | 50% | 75% | 100% |
| *w* | 2090.00 | 7949.50 | 11024.50 | 14004.00 | 16246.00 |
| AD | 20.00 | 122.75 | 983.50 | 2097.00 | 20019.00 |
| WAD | 40.00 | 428.25 | 1928.00 | 3287.25 | 19185.00 |
| FC | 18.00 | 122.75 | 910.50 | 2067.25 | 19907.00 |
| RP | 27.00 | 111.25 | 577.00 | 2378.25 | 21987.00 |
| modT | 16.00 | 157.75 | 1862.00 | 5322.00 | 19637.00 |
| samT | 26.00 | 342.00 | 2972.00 | 7551.00 | 19196.00 |
| shrinkT | 16.00 | 180.25 | 2035.00 | 5722.75 | 19558.00 |
| ibmT | 18.00 | 122.75 | 1652.50 | 5085.00 | 19784.00 |
| DFW-preprocessed data | | |  |  |  |
| Method | 0% | 25% | 50% | 75% | 100% |
| *w* | 4693.00 | 10117.75 | 18438.50 | 19934.50 | 21447.00 |
| AD | 58.00 | 134.00 | 770.00 | 3509.00 | 20850.00 |
| WAD | 102.00 | 196.50 | 1010.50 | 3434.00 | 20559.00 |
| FC | 58.00 | 134.00 | 769.00 | 3509.50 | 20850.00 |
| RP | 75.00 | 148.25 | 570.00 | 4663.25 | 20931.00 |
| modT | 64.00 | 730.75 | 3975.00 | 6282.00 | 19557.00 |
| samT | 65.00 | 744.50 | 4178.50 | 6685.25 | 17869.00 |
| shrinkT | 63.00 | 682.50 | 3125.00 | 4678.00 | 20734.00 |
| ibmT | 64.00 | 725.50 | 3971.00 | 6344.50 | 19663.00 |

**8-2.**

| Method | MAS | RMA | DFW |
| --- | --- | --- | --- |
| *w* | 0.58103 | 0.51325 | 0.32795 |
| AD | 0.82812 | 0.89021 | 0.88503 |
| WAD | 0.87211 | 0.85745 | 0.86785 |
| FC | 0.83046 | 0.89239 | 0.88503 |
| RP | 0.84582 | 0.88116 | 0.87302 |
| modT | 0.83172 | 0.82843 | 0.77304 |
| samT | 0.83969 | 0.78462 | 0.76772 |
| shrinkT | 0.83589 | 0.82075 | 0.81062 |
| ibmT | 0.84848 | 0.83495 | 0.77192 |

**Dataset 10**

**1.** http://www.ncbi.nlm.nih.gov/sites/entrez?Db=Pubmed&term=15381369[UID]

**2.** GSE3524

**3.** 16 OSCE:

GSM80460-80475

**4.** 4 normal:

GSM80476, GSM80477, GSM80520, GSM80521

**5.** 4 probesets.

"201105_at", "204475_at", "211597_s_at", "213240_s_at"

**6.** MAS5.0

**7.** RMA- and DFW-preprocessed data were obtained from a total of 20 samples in GSE3524.

**8-1.**

| MAS-preprocessed data | | |  |  |  |
| --- | --- | --- | --- | --- | --- |
| Method | 0% | 25% | 50% | 75% | 100% |
| *w* | 1139.00 | 1454.75 | 3623.00 | 6334.00 | 8278.00 |
| AD | 2.00 | 16.25 | 22.50 | 26.50 | 34.00 |
| WAD | 2.00 | 10.25 | 23.50 | 35.25 | 39.00 |
| FC | 21.00 | 23.25 | 29.00 | 41.50 | 64.00 |
| RP | 4.00 | 13.75 | 20.00 | 26.00 | 35.00 |
| modT | 30.00 | 130.50 | 232.00 | 301.00 | 304.00 |
| samT | 21.00 | 63.00 | 89.00 | 109.00 | 133.00 |
| shrinkT | 26.00 | 107.00 | 190.00 | 247.00 | 250.00 |
| ibmT | 28.00 | 123.25 | 211.50 | 272.25 | 285.00 |
| RMA-preprocessed data | | |  |  |  |
| Method | 0% | 25% | 50% | 75% | 100% |
| *w* | 440.00 | 1337.00 | 3644.00 | 6096.75 | 7431.00 |
| AD | 3.00 | 5.25 | 11.00 | 34.25 | 89.00 |
| WAD | 2.00 | 8.00 | 49.00 | 111.25 | 181.00 |
| FC | 5.00 | 10.25 | 15.50 | 27.75 | 54.00 |
| RP | 4.00 | 7.00 | 12.00 | 35.50 | 94.00 |
| modT | 87.00 | 198.00 | 1128.00 | 2059.75 | 2176.00 |
| samT | 71.00 | 164.75 | 988.50 | 1817.50 | 1927.00 |
| shrinkT | 64.00 | 151.00 | 894.00 | 1638.75 | 1731.00 |
| ibmT | 73.00 | 168.25 | 1057.50 | 1953.00 | 2067.00 |
| DFW-preprocessed data | | |  |  |  |
| Method | 0% | 25% | 50% | 75% | 100% |
| *w* | 354.00 | 4113.00 | 5792.50 | 8143.75 | 13918.00 |
| AD | 1.00 | 16.00 | 23.00 | 45.75 | 108.00 |
| WAD | 2.00 | 28.25 | 40.50 | 60.00 | 108.00 |
| FC | 1.00 | 13.00 | 21.00 | 45.50 | 107.00 |
| RP | 1.00 | 7.75 | 13.00 | 33.25 | 85.00 |
| modT | 34.00 | 183.25 | 1757.50 | 3646.25 | 4739.00 |
| samT | 31.00 | 175.75 | 1721.50 | 3588.25 | 4696.00 |
| shrinkT | 27.00 | 121.50 | 1136.50 | 2404.25 | 3257.00 |
| ibmT | 30.00 | 175.50 | 1764.00 | 3669.75 | 4767.00 |

**8-2.**

| Method | MAS | RMA | DFW |
| --- | --- | --- | --- |
| *w* | 0.81313 | 0.83001 | 0.70996 |
| AD | 0.99920 | 0.99883 | 0.99837 |
| WAD | 0.99912 | 0.99696 | 0.99797 |
| FC | 0.99851 | 0.99910 | 0.99843 |
| RP | 0.99923 | 0.99874 | 0.99886 |
| modT | 0.99116 | 0.94940 | 0.90711 |
| samT | 0.99639 | 0.95551 | 0.90843 |
| shrinkT | 0.99275 | 0.95991 | 0.93776 |
| ibmT | 0.99185 | 0.95237 | 0.90669 |

**Dataset 11**

**1.** http://www.ncbi.nlm.nih.gov/sites/entrez?Db=Pubmed&term=15268757[UID]

**2.** GSE3860

**3.** 9 HGPS:

GSM87553, GSM87751, GSM88282-88288

**4.** 9 control:

GSM88289-88297

**5.** 8 probesets.

"206201_s_at", "201858_s_at", "201596_x_at", "210809_s_at", "200887_s_at",

"218899_s_at", "210239_at", "205830_at"

**6.** MAS5.0

**7.** RMA- and DFW-preprocessed data were obtained from a total of 18 samples in GSE3860.

**8-1.**

| MAS-preprocessed data | | |  |  |  |
| --- | --- | --- | --- | --- | --- |
| Method | 0% | 25% | 50% | 75% | 100% |
| *w* | 934.00 | 1530.25 | 6393.50 | 11957.00 | 15726.00 |
| AD | 1.00 | 2.75 | 32.00 | 52.25 | 361.00 |
| WAD | 1.00 | 2.75 | 25.50 | 65.75 | 185.00 |
| FC | 6.00 | 22.00 | 57.50 | 91.75 | 337.00 |
| RP | 1.00 | 2.75 | 29.50 | 48.25 | 376.00 |
| modT | 17.00 | 39.00 | 42.50 | 63.25 | 492.00 |
| samT | 1.00 | 14.25 | 30.00 | 52.50 | 248.00 |
| shrinkT | 13.00 | 39.00 | 42.50 | 61.75 | 446.00 |
| ibmT | 17.00 | 32.00 | 44.00 | 60.50 | 464.00 |
| RMA-preprocessed data | | |  |  |  |
| Method | 0% | 25% | 50% | 75% | 100% |
| *w* | 963.00 | 1409.75 | 6156.50 | 13565.50 | 20009.00 |
| AD | 1.00 | 4.50 | 22.50 | 101.25 | 141.00 |
| WAD | 1.00 | 2.75 | 46.50 | 144.25 | 1792.00 |
| FC | 1.00 | 7.25 | 52.50 | 102.00 | 116.00 |
| RP | 2.00 | 6.00 | 20.00 | 60.25 | 105.00 |
| modT | 2.00 | 6.75 | 44.50 | 155.75 | 709.00 |
| samT | 2.00 | 6.75 | 41.50 | 147.75 | 671.00 |
| shrinkT | 2.00 | 6.75 | 46.00 | 161.00 | 742.00 |
| ibmT | 2.00 | 6.75 | 38.50 | 138.50 | 657.00 |
| DFW-preprocessed data | | |  |  |  |
| Method | 0% | 25% | 50% | 75% | 100% |
| *w* | 774.00 | 1948.75 | 9414.00 | 18231.00 | 22048.00 |
| AD | 1.00 | 2.75 | 67.00 | 213.50 | 727.00 |
| WAD | 1.00 | 2.75 | 97.00 | 299.00 | 1695.00 |
| FC | 1.00 | 2.75 | 66.00 | 213.00 | 728.00 |
| RP | 1.00 | 3.75 | 47.00 | 115.25 | 421.00 |
| modT | 2.00 | 28.00 | 45.50 | 186.00 | 631.00 |
| samT | 2.00 | 27.25 | 45.50 | 184.25 | 627.00 |
| shrinkT | 2.00 | 27.25 | 44.50 | 178.75 | 606.00 |
| ibmT | 2.00 | 27.25 | 45.00 | 184.00 | 622.00 |

**8-2.**

| Method | MAS | RMA | DFW |
| --- | --- | --- | --- |
| *w* | 0.67461 | 0.64044 | 0.52735 |
| AD | 0.99715 | 0.99793 | 0.99219 |
| WAD | 0.99792 | 0.98749 | 0.98510 |
| FC | 0.99616 | 0.99773 | 0.99221 |
| RP | 0.99715 | 0.99852 | 0.99557 |
| modT | 0.99562 | 0.99277 | 0.99244 |
| samT | 0.99769 | 0.99318 | 0.99250 |
| shrinkT | 0.99593 | 0.99246 | 0.99273 |
| ibmT | 0.99589 | 0.99348 | 0.99254 |

**Dataset 12**

**1.** http://www.ncbi.nlm.nih.gov/sites/entrez?Db=Pubmed&term=17181634[UID]

**2.** GSE5667

**3.** 5 control:

GSM132623-132627

**4.** 6 NLAD:

GSM132628-132633

**5.** 3 probesets.

"206407_s_at", "211338_at", "212592_at"

**6.** MAS5.0

**7.** RMA- and DFW-preprocessed data were obtained from a total of **17** samples in GSE5667.

**8-1.**

| MAS-preprocessed data | | |  |  |  |
| --- | --- | --- | --- | --- | --- |
| Method | 0% | 25% | 50% | 75% | 100% |
| *w* | 4942.00 | 11190.00 | 17438.00 | 19573.50 | 21709.00 |
| AD | 5.00 | 9.00 | 13.00 | 143.00 | 273.00 |
| WAD | 15.00 | 27.00 | 39.00 | 224.50 | 410.00 |
| FC | 8.00 | 12.00 | 16.00 | 103.00 | 190.00 |
| RP | 8.00 | 15.50 | 23.00 | 188.00 | 353.00 |
| modT | 14.00 | 48.00 | 82.00 | 93.50 | 105.00 |
| samT | 17.00 | 55.50 | 94.00 | 112.00 | 130.00 |
| shrinkT | 12.00 | 43.00 | 74.00 | 85.50 | 97.00 |
| ibmT | 5.00 | 16.50 | 28.00 | 32.50 | 37.00 |
| RMA-preprocessed data | | |  |  |  |
| Method | 0% | 25% | 50% | 75% | 100% |
| *w* | 6179.00 | 13306.50 | 20434.00 | 20532.50 | 20631.00 |
| AD | 14.00 | 31.00 | 48.00 | 7097.50 | 14147.00 |
| WAD | 22.00 | 1044.00 | 2066.00 | 10363.00 | 18660.00 |
| FC | 22.00 | 27.00 | 32.00 | 7285.00 | 14538.00 |
| RP | 9.00 | 46.00 | 83.00 | 9729.00 | 19375.00 |
| modT | 15.00 | 105.50 | 196.00 | 4788.00 | 9380.00 |
| samT | 3.00 | 46.00 | 89.00 | 4738.00 | 9387.00 |
| shrinkT | 25.00 | 136.50 | 248.00 | 4416.00 | 8584.00 |
| ibmT | 10.00 | 72.50 | 135.00 | 3625.50 | 7116.00 |
| DFW-preprocessed data | | |  |  |  |
| Method | 0% | 25% | 50% | 75% | 100% |
| *w* | 8152.00 | 14452.50 | 20753.00 | 21037.00 | 21321.00 |
| AD | 55.00 | 89.00 | 123.00 | 8964.00 | 17805.00 |
| WAD | 56.00 | 174.00 | 292.00 | 9835.50 | 19379.00 |
| FC | 55.00 | 90.00 | 125.00 | 8965.00 | 17805.00 |
| RP | 6.00 | 28.00 | 50.00 | 10294.50 | 20539.00 |
| modT | 81.00 | 248.50 | 416.00 | 3977.00 | 7538.00 |
| samT | 89.00 | 261.00 | 433.00 | 2861.50 | 5290.00 |
| shrinkT | 39.00 | 134.50 | 230.00 | 7454.50 | 14679.00 |
| ibmT | 79.00 | 237.50 | 396.00 | 2653.50 | 4911.00 |

**8-2.**

| Method | MAS | RMA | DFW |
| --- | --- | --- | --- |
| *w* | 0.34047 | 0.29327 | 0.24865 |
| AD | 0.99574 | 0.78751 | 0.73104 |
| WAD | 0.99315 | 0.68968 | 0.70495 |
| FC | 0.99689 | 0.78178 | 0.73101 |
| RP | 0.99434 | 0.70884 | 0.69197 |
| modT | 0.99708 | 0.85660 | 0.87988 |
| samT | 0.99648 | 0.85827 | 0.91314 |
| shrinkT | 0.99735 | 0.86758 | 0.77645 |
| ibmT | 0.99904 | 0.89146 | 0.91951 |

**Dataset 13**

**1.** http://www.ncbi.nlm.nih.gov/sites/entrez?Db=Pubmed&term=17181634[UID]

**2.** GSE5667

**3.** 5 control:

GSM132623-132627

**4.** 6 LAD:

GSM132634-13639

**5.** 3 probesets.

"206407_s_at", "211338_at", "212592_at"

**6.** MAS5.0

**7.** RMA- and DFW-preprocessed data were obtained from a total of **17** samples in GSE5667.

**8-1.**

| MAS-preprocessed data | | |  |  |  |
| --- | --- | --- | --- | --- | --- |
| Method | 0% | 25% | 50% | 75% | 100% |
| *w* | 3364.00 | 9570.50 | 15777.00 | 18543.00 | 21309.00 |
| AD | 58.00 | 157.00 | 256.00 | 295.50 | 335.00 |
| WAD | 32.00 | 186.50 | 341.00 | 1525.00 | 2709.00 |
| FC | 63.00 | 92.50 | 122.00 | 292.50 | 463.00 |
| RP | 69.00 | 192.50 | 316.00 | 335.50 | 355.00 |
| modT | 2.00 | 1251.00 | 2500.00 | 2551.00 | 2602.00 |
| samT | 3.00 | 857.50 | 1712.00 | 1773.50 | 1835.00 |
| shrinkT | 2.00 | 1166.50 | 2331.00 | 2388.00 | 2445.00 |
| ibmT | 2.00 | 1144.50 | 2287.00 | 2402.00 | 2517.00 |
| RMA-preprocessed data | | |  |  |  |
| Method | 0% | 25% | 50% | 75% | 100% |
| *w* | 4214.00 | 12253.50 | 20293.00 | 20426.50 | 20560.00 |
| AD | 19.00 | 196.00 | 373.00 | 9335.50 | 18298.00 |
| WAD | 25.00 | 2693.00 | 5361.00 | 12721.50 | 20082.00 |
| FC | 22.00 | 105.50 | 189.00 | 8980.00 | 17771.00 |
| RP | 14.00 | 310.50 | 607.00 | 10795.50 | 20984.00 |
| modT | 3.00 | 1724.00 | 3445.00 | 10302.00 | 17159.00 |
| samT | 3.00 | 1800.00 | 3597.00 | 10257.50 | 16918.00 |
| shrinkT | 3.00 | 1798.50 | 3594.00 | 10334.00 | 17074.00 |
| ibmT | 3.00 | 1543.00 | 3083.00 | 9740.00 | 16397.00 |
| DFW-preprocessed data | | |  |  |  |
| Method | 0% | 25% | 50% | 75% | 100% |
| *w* | 8009.00 | 14381.00 | 20753.00 | 21036.50 | 21320.00 |
| AD | 57.00 | 147.00 | 237.00 | 10726.50 | 21216.00 |
| WAD | 59.00 | 315.00 | 571.00 | 11058.50 | 21546.00 |
| FC | 57.00 | 146.50 | 236.00 | 10726.00 | 21216.00 |
| RP | 28.00 | 257.00 | 486.00 | 11174.50 | 21863.00 |
| modT | 32.00 | 2913.50 | 5795.00 | 12656.50 | 19518.00 |
| samT | 29.00 | 2942.00 | 5855.00 | 12555.50 | 19256.00 |
| shrinkT | 22.00 | 2202.50 | 4383.00 | 12585.00 | 20787.00 |
| ibmT | 30.00 | 2873.50 | 5717.00 | 12360.00 | 19003.00 |

**8-2.**

| Method | MAS | RMA | DFW |
| --- | --- | --- | --- |
| *w* | 0.39491 | 0.32584 | 0.25081 |
| AD | 0.99038 | 0.72047 | 0.67828 |
| WAD | 0.95398 | 0.61906 | 0.66831 |
| FC | 0.99039 | 0.73106 | 0.67829 |
| RP | 0.98902 | 0.67686 | 0.66531 |
| modT | 0.92373 | 0.69179 | 0.62090 |
| samT | 0.94698 | 0.69312 | 0.62397 |
| shrinkT | 0.92861 | 0.69083 | 0.62319 |
| ibmT | 0.92819 | 0.70860 | 0.62980 |

**Dataset 14**

**1.** http://www.ncbi.nlm.nih.gov/sites/entrez?Db=Pubmed&term=17405831[UID]

**2.** GSE6236

**3.** 14 AB:

GSM143572-143585

**4.** 14 CB:

GSM143586-143599

**5.** 7 probesets.

"219630_at", "203388_at", "207854_at", "205838_at", "211935_at",

"217845_x_at", "221896_s_at"

**6.** MAS5.0

**7.** RMA- and DFW-preprocessed data were obtained from a total of 28 samples in GSE6236.

**8-1.**

| MAS-preprocessed data | | |  |  |  |
| --- | --- | --- | --- | --- | --- |
| Method | 0% | 25% | 50% | 75% | 100% |
| *w* | 444.00 | 901.50 | 1340.00 | 2494.50 | 5145.00 |
| AD | 1.00 | 8.00 | 24.00 | 141.50 | 197.00 |
| WAD | 1.00 | 11.50 | 27.00 | 106.00 | 140.00 |
| FC | 1.00 | 7.50 | 17.00 | 125.00 | 187.00 |
| RP | 1.00 | 5.00 | 23.00 | 150.50 | 194.00 |
| modT | 7.00 | 10.50 | 20.00 | 82.50 | 169.00 |
| samT | 2.00 | 5.50 | 8.00 | 87.50 | 166.00 |
| shrinkT | 3.00 | 8.50 | 13.00 | 81.50 | 153.00 |
| ibmT | 14.00 | 15.50 | 25.00 | 85.00 | 162.00 |
| RMA-preprocessed data | | |  |  |  |
| Method | 0% | 25% | 50% | 75% | 100% |
| *w* | 309.00 | 2840.00 | 5850.00 | 9598.50 | 13174.00 |
| AD | 2.00 | 26.50 | 54.00 | 111.00 | 259.00 |
| WAD | 11.00 | 61.50 | 114.00 | 185.00 | 335.00 |
| FC | 1.00 | 28.00 | 53.00 | 109.50 | 278.00 |
| RP | 1.00 | 6.00 | 32.00 | 82.50 | 151.00 |
| modT | 1.00 | 17.00 | 44.00 | 126.00 | 163.00 |
| samT | 1.00 | 17.00 | 40.00 | 126.00 | 164.00 |
| shrinkT | 1.00 | 17.00 | 45.00 | 127.50 | 169.00 |
| ibmT | 1.00 | 18.00 | 41.00 | 120.50 | 159.00 |
| DFW-preprocessed data | | |  |  |  |
| Method | 0% | 25% | 50% | 75% | 100% |
| *w* | 706.00 | 7664.00 | 16306.00 | 17272.00 | 18488.00 |
| AD | 36.00 | 73.50 | 115.00 | 189.50 | 351.00 |
| WAD | 65.00 | 109.00 | 175.00 | 254.50 | 361.00 |
| FC | 37.00 | 73.00 | 115.00 | 189.50 | 351.00 |
| RP | 3.00 | 60.50 | 110.00 | 162.50 | 314.00 |
| modT | 11.00 | 33.50 | 59.00 | 89.50 | 110.00 |
| samT | 11.00 | 33.50 | 59.00 | 89.50 | 110.00 |
| shrinkT | 11.00 | 33.50 | 59.00 | 89.50 | 110.00 |
| ibmT | 11.00 | 33.50 | 59.00 | 89.50 | 110.00 |

**8-2.**

| Method | MAS | RMA | DFW |
| --- | --- | --- | --- |
| *w* | 0.91219 | 0.71666 | 0.45268 |
| AD | 0.99684 | 0.99640 | 0.99359 |
| WAD | 0.99760 | 0.99407 | 0.99166 |
| FC | 0.99717 | 0.99629 | 0.99359 |
| RP | 0.99679 | 0.99786 | 0.99458 |
| modT | 0.99773 | 0.99701 | 0.99745 |
| samT | 0.99786 | 0.99703 | 0.99745 |
| shrinkT | 0.99794 | 0.99695 | 0.99745 |
| ibmT | 0.99760 | 0.99711 | 0.99745 |

**Dataset 15**

**1.** http://www.ncbi.nlm.nih.gov/sites/entrez?Db=Pubmed&term=17699851[UID]

**2.** GSE6344

**3.** 10 normal samples:

GSM146778, GSM146780, GSM146782, GSM146784, GSM146786,

GSM146789, GSM146790, GSM146792, GSM146794, GSM146796

**4.** 10 tumor samples:

GSM146779, GSM146781, GSM146783, GSM146785, GSM146787,

GSM146788, GSM146791, GSM146793, GSM146795, GSM146797

**5.** 19 probesets.

"202035_s_at", "202036_s_at", "202037_s_at", "208711_s_at", "208712_at",

"202431_s_at", "201667_at", "218995_s_at", "210495_x_at", "211719_x_at",

"212464_s_at", "216442_x_at", "201426_s_at", "200665_s_at", "201666_at",

"210512_s_at", "210513_s_at", "211527_x_at", "212171_x_at"

**6.** MAS5.0

**7.** RMA- and DFW-preprocessed data were obtained from a total of 20 samples in GSE6344.

**8-1.**

| MAS-preprocessed data | | |  |  |  |
| --- | --- | --- | --- | --- | --- |
| Method | 0% | 25% | 50% | 75% | 100% |
| *w* | 142.00 | 711.00 | 1655.00 | 3452.50 | 11197.00 |
| AD | 21.00 | 266.00 | 452.00 | 907.50 | 1116.00 |
| WAD | 15.00 | 108.00 | 230.00 | 308.50 | 609.00 |
| FC | 23.00 | 272.50 | 462.00 | 767.50 | 1175.00 |
| RP | 24.00 | 237.50 | 385.00 | 843.00 | 1052.00 |
| modT | 4.00 | 160.50 | 250.00 | 781.50 | 2022.00 |
| samT | 11.00 | 177.00 | 302.00 | 533.50 | 962.00 |
| shrinkT | 4.00 | 150.50 | 220.00 | 715.50 | 1799.00 |
| ibmT | 2.00 | 138.50 | 223.00 | 743.50 | 1872.00 |
| RMA-preprocessed data | | |  |  |  |
| Method | 0% | 25% | 50% | 75% | 100% |
| *w* | 136.00 | 772.50 | 2081.00 | 3436.50 | 12631.00 |
| AD | 15.00 | 83.00 | 174.00 | 239.00 | 520.00 |
| WAD | 16.00 | 38.50 | 93.00 | 168.00 | 487.00 |
| FC | 17.00 | 88.00 | 188.00 | 271.50 | 426.00 |
| RP | 15.00 | 84.00 | 171.00 | 256.50 | 597.00 |
| modT | 6.00 | 118.50 | 239.00 | 585.00 | 1918.00 |
| samT | 5.00 | 113.50 | 219.00 | 511.00 | 1681.00 |
| shrinkT | 5.00 | 115.50 | 231.00 | 549.50 | 1806.00 |
| ibmT | 5.00 | 118.00 | 220.00 | 528.00 | 1763.00 |
| DFW-preprocessed data | | |  |  |  |
| Method | 0% | 25% | 50% | 75% | 100% |
| *w* | 245.00 | 1651.50 | 3618.00 | 6680.00 | 21688.00 |
| AD | 32.00 | 70.50 | 119.00 | 185.00 | 351.00 |
| WAD | 39.00 | 54.50 | 100.00 | 176.00 | 697.00 |
| FC | 33.00 | 71.50 | 118.00 | 185.00 | 352.00 |
| RP | 29.00 | 50.50 | 104.00 | 155.00 | 327.00 |
| modT | 7.00 | 164.50 | 353.00 | 920.50 | 2028.00 |
| samT | 7.00 | 163.50 | 352.00 | 913.50 | 2008.00 |
| shrinkT | 7.00 | 160.50 | 333.00 | 846.00 | 1806.00 |
| ibmT | 7.00 | 164.50 | 350.00 | 909.00 | 2017.00 |

**8-2.**

| Method | MAS | RMA | DFW |
| --- | --- | --- | --- |
| *w* | 0.88729 | 0.87757 | 0.76024 |
| AD | 0.97647 | 0.99227 | 0.99429 |
| WAD | 0.99029 | 0.99392 | 0.99347 |
| FC | 0.97763 | 0.99199 | 0.99424 |
| RP | 0.97839 | 0.99162 | 0.99520 |
| modT | 0.97896 | 0.98117 | 0.97425 |
| samT | 0.98379 | 0.98367 | 0.97444 |
| shrinkT | 0.98077 | 0.98216 | 0.97637 |
| ibmT | 0.98060 | 0.98288 | 0.97449 |

**Dataset 16**

**1.** http://www.ncbi.nlm.nih.gov/sites/entrez?Db=Pubmed&term=16858420[UID]

**2.** GSE6710

**3.** 13 lesional skin samples:

GSM154768, GSM154770, GSM154772, GSM154774, GSM154776,

GSM154778, GSM154780, GSM154782, GSM154784, GSM154786,

GSM154788, GSM154790, GSM154792

**4.** 13 uninvolved skin samples:

GSM154769, GSM154771, GSM154773, GSM154775, GSM154777,

GSM154779, GSM154781, GSM154783, GSM154785, GSM154787,

GSM154789, GSM154791, GSM154793

**5.** 7 probesets.

"205990_s_at", "213425_at", "209800_at", "203697_at", "203698_s_at",

"219908_at", "208712_at"

**6.** MAS5.0

**7.** RMA- and DFW-preprocessed data were obtained from a total of 26 samples in GSE6710.

**8-1.**

| MAS-preprocessed data | | |  |  |  |
| --- | --- | --- | --- | --- | --- |
| Method | 0% | 25% | 50% | 75% | 100% |
| *w* | 967.00 | 4650.50 | 7824.00 | 8787.50 | 11581.00 |
| AD | 30.00 | 322.50 | 494.00 | 769.00 | 995.00 |
| WAD | 12.00 | 294.50 | 444.00 | 496.00 | 668.00 |
| FC | 46.00 | 310.00 | 480.00 | 759.50 | 818.00 |
| RP | 34.00 | 347.50 | 540.00 | 658.00 | 840.00 |
| modT | 45.00 | 136.00 | 268.00 | 391.00 | 795.00 |
| samT | 45.00 | 106.00 | 238.00 | 400.50 | 571.00 |
| shrinkT | 46.00 | 122.00 | 246.00 | 366.00 | 728.00 |
| ibmT | 49.00 | 147.50 | 279.00 | 376.50 | 776.00 |
| RMA-preprocessed data | | |  |  |  |
| Method | 0% | 25% | 50% | 75% | 100% |
| *w* | 920.00 | 4643.50 | 8901.00 | 10908.00 | 18209.00 |
| AD | 9.00 | 128.00 | 223.00 | 318.50 | 606.00 |
| WAD | 5.00 | 272.50 | 437.00 | 713.50 | 3056.00 |
| FC | 16.00 | 129.00 | 221.00 | 322.50 | 580.00 |
| RP | 10.00 | 104.00 | 192.00 | 304.00 | 537.00 |
| modT | 58.00 | 211.50 | 315.00 | 515.00 | 849.00 |
| samT | 59.00 | 218.00 | 324.00 | 534.00 | 872.00 |
| shrinkT | 57.00 | 199.00 | 303.00 | 496.50 | 820.00 |
| ibmT | 56.00 | 187.50 | 280.00 | 493.00 | 783.00 |
| DFW-preprocessed data | | |  |  |  |
| Method | 0% | 25% | 50% | 75% | 100% |
| *w* | 3141.00 | 9869.00 | 15556.00 | 18973.00 | 19284.00 |
| AD | 5.00 | 400.50 | 619.00 | 662.50 | 1638.00 |
| WAD | 6.00 | 572.50 | 830.00 | 1049.00 | 2549.00 |
| FC | 5.00 | 400.00 | 620.00 | 662.50 | 1636.00 |
| RP | 5.00 | 294.50 | 460.00 | 515.50 | 1100.00 |
| modT | 31.00 | 164.50 | 477.00 | 700.00 | 883.00 |
| samT | 31.00 | 165.00 | 480.00 | 704.50 | 888.00 |
| shrinkT | 31.00 | 162.50 | 467.00 | 680.00 | 845.00 |
| ibmT | 31.00 | 163.50 | 476.00 | 698.00 | 875.00 |

**8-2.**

| Method | MAS | RMA | DFW |
| --- | --- | --- | --- |
| *w* | 0.69718 | 0.62096 | 0.38667 |
| AD | 0.97644 | 0.98908 | 0.97204 |
| WAD | 0.98283 | 0.96510 | 0.95767 |
| FC | 0.97784 | 0.98915 | 0.97205 |
| RP | 0.97821 | 0.99021 | 0.97975 |
| modT | 0.98631 | 0.98302 | 0.98017 |
| samT | 0.98821 | 0.98249 | 0.98006 |
| shrinkT | 0.98738 | 0.98369 | 0.98076 |
| ibmT | 0.98638 | 0.98428 | 0.98027 |

**Dataset 17**

**1.** http://www.ncbi.nlm.nih.gov/sites/entrez?Db=Pubmed&term=17472435[UID]

**2.** GSE7146

**3.** 6 pre-clamp samples:

GSM172123, GSM172129, GSM172131, GSM172133, GSM172136,

GSM172140

**4.** 6 post-clamp samples:

GSM172125, GSM172130, GSM172132, GSM172134, GSM172138,

GSM172142

**5.** 6 probesets.

"213524_s_at", "203140_at", "215990_s_at", "201008_s_at", "201009_s_at",

"201010_s_at"

**6.** MAS5.0

**7.** RMA- and DFW-preprocessed data were obtained from a total of **12** samples in GSE7146.

**8-1.**

| MAS-preprocessed data | | |  |  |  |
| --- | --- | --- | --- | --- | --- |
| Method | 0% | 25% | 50% | 75% | 100% |
| *w* | 291.00 | 384.25 | 759.50 | 1234.50 | 11035.00 |
| AD | 157.00 | 694.25 | 775.00 | 1224.75 | 1865.00 |
| WAD | 1.00 | 3.00 | 7.50 | 56.25 | 276.00 |
| FC | 407.00 | 570.75 | 737.50 | 984.50 | 2050.00 |
| RP | 429.00 | 830.75 | 1003.00 | 1555.50 | 2464.00 |
| modT | 29.00 | 41.50 | 101.50 | 147.25 | 258.00 |
| samT | 25.00 | 48.25 | 88.50 | 156.50 | 210.00 |
| shrinkT | 27.00 | 46.00 | 125.00 | 171.75 | 320.00 |
| ibmT | 3.00 | 8.25 | 24.00 | 49.50 | 101.00 |
| RMA-preprocessed data | | |  |  |  |
| Method | 0% | 25% | 50% | 75% | 100% |
| *w* | 213.00 | 384.50 | 970.00 | 1393.50 | 4501.00 |
| AD | 1.00 | 2.75 | 6.00 | 13.00 | 11918.00 |
| WAD | 1.00 | 2.25 | 3.50 | 14.50 | 7796.00 |
| FC | 1.00 | 2.75 | 6.50 | 19.25 | 9492.00 |
| RP | 1.00 | 3.00 | 7.00 | 16.25 | 10380.00 |
| modT | 1.00 | 14.75 | 40.50 | 109.75 | 13591.00 |
| samT | 1.00 | 8.00 | 17.50 | 42.00 | 13401.00 |
| shrinkT | 1.00 | 24.75 | 70.50 | 165.00 | 13790.00 |
| ibmT | 1.00 | 10.25 | 28.00 | 78.75 | 13742.00 |
| DFW-preprocessed data | | |  |  |  |
| Method | 0% | 25% | 50% | 75% | 100% |
| *w* | 408.00 | 489.25 | 1897.50 | 3230.00 | 4147.00 |
| AD | 2.00 | 7.00 | 22.50 | 118.25 | 5949.00 |
| WAD | 1.00 | 7.25 | 35.00 | 206.00 | 5009.00 |
| FC | 1.00 | 6.25 | 22.00 | 117.25 | 5950.00 |
| RP | 1.00 | 2.25 | 3.50 | 27.25 | 6812.00 |
| modT | 3.00 | 34.75 | 98.50 | 258.25 | 5112.00 |
| samT | 4.00 | 41.25 | 116.00 | 288.25 | 5373.00 |
| shrinkT | 2.00 | 21.50 | 55.00 | 162.75 | 4330.00 |
| ibmT | 2.00 | 28.75 | 79.50 | 227.75 | 5177.00 |

**8-2.**

| Method | MAS | RMA | DFW |
| --- | --- | --- | --- |
| *w* | 0.89170 | 0.93748 | 0.91005 |
| AD | 0.95816 | 0.91077 | 0.95416 |
| WAD | 0.99742 | 0.94162 | 0.96020 |
| FC | 0.95890 | 0.92885 | 0.95418 |
| RP | 0.94470 | 0.92223 | 0.94886 |
| modT | 0.99511 | 0.89682 | 0.95798 |
| samT | 0.99547 | 0.89922 | 0.95548 |
| shrinkT | 0.99414 | 0.89438 | 0.96536 |
| ibmT | 0.99856 | 0.89617 | 0.95806 |

**Dataset 18**

**1.** http://www.ncbi.nlm.nih.gov/sites/entrez?Db=Pubmed&term=17517823[UID]

**2.** GSE7765

**3.** 3 Dioxin-treated samples:

GSM188014, GSM188018, GSM188022

**4.** 3 control (DMSO-treated) samples:

GSM188013, GSM188016, GSM188020

**5.** 13 probesets.

"205623_at", "202887_s_at", "218729_at", "204341_at", "209201_x_at",

"217028_at", "218559_s_at", "204285_s_at", "204286_s_at", "206115_at",

"203666_at", "209687_at", "217763_s_at"

**6.** MAS5.0

**7.** RMA- and DFW-preprocessed data were obtained from a total of **6** samples in GSE7765.

**8-1.**

| MAS-preprocessed data | | |  |  |  |
| --- | --- | --- | --- | --- | --- |
| Method | 0% | 25% | 50% | 75% | 100% |
| *w* | 557.00 | 2733.00 | 6740.00 | 10775.00 | 13456.00 |
| AD | 4.00 | 624.00 | 810.00 | 1478.00 | 3186.00 |
| WAD | 8.00 | 18.00 | 111.00 | 256.00 | 1813.00 |
| FC | 7.00 | 683.00 | 1090.00 | 1428.00 | 3555.00 |
| RP | 4.00 | 510.00 | 778.00 | 1365.00 | 4217.00 |
| modT | 2.00 | 68.00 | 212.00 | 571.00 | 2655.00 |
| samT | 3.00 | 79.00 | 220.00 | 524.00 | 2690.00 |
| shrinkT | 2.00 | 61.00 | 219.00 | 599.00 | 2730.00 |
| ibmT | 5.00 | 39.00 | 91.00 | 248.00 | 2250.00 |
| RMA-preprocessed data | | |  |  |  |
| Method | 0% | 25% | 50% | 75% | 100% |
| *w* | 627.00 | 3083.00 | 7674.00 | 10041.00 | 19520.00 |
| AD | 5.00 | 28.00 | 64.00 | 84.00 | 784.00 |
| WAD | 7.00 | 35.00 | 55.00 | 663.00 | 5621.00 |
| FC | 4.00 | 35.00 | 72.00 | 87.00 | 957.00 |
| RP | 5.00 | 25.00 | 63.00 | 77.00 | 726.00 |
| modT | 12.00 | 43.00 | 187.00 | 262.00 | 2065.00 |
| samT | 6.00 | 38.00 | 107.00 | 153.00 | 1370.00 |
| shrinkT | 12.00 | 48.00 | 247.00 | 370.00 | 2613.00 |
| ibmT | 7.00 | 27.00 | 139.00 | 196.00 | 2023.00 |
| DFW-preprocessed data | | |  |  |  |
| Method | 0% | 25% | 50% | 75% | 100% |
| *w* | 1732.00 | 3940.00 | 9764.00 | 15815.00 | 21552.00 |
| AD | 16.00 | 35.00 | 50.00 | 444.00 | 2924.00 |
| WAD | 15.00 | 30.00 | 69.00 | 1177.00 | 3691.00 |
| FC | 16.00 | 35.00 | 50.00 | 443.00 | 2924.00 |
| RP | 11.00 | 22.00 | 35.00 | 285.00 | 2344.00 |
| modT | 36.00 | 105.00 | 379.00 | 676.00 | 1496.00 |
| samT | 34.00 | 108.00 | 362.00 | 657.00 | 1442.00 |
| shrinkT | 27.00 | 76.00 | 213.00 | 362.00 | 737.00 |
| ibmT | 32.00 | 98.00 | 358.00 | 647.00 | 1482.00 |

**8-2.**

| Method | MAS | RMA | DFW |
| --- | --- | --- | --- |
| *w* | 0.67609 | 0.62088 | 0.51240 |
| AD | 0.95202 | 0.99392 | 0.97873 |
| WAD | 0.98781 | 0.96936 | 0.96252 |
| FC | 0.95019 | 0.99287 | 0.97873 |
| RP | 0.95240 | 0.99382 | 0.98498 |
| modT | 0.97906 | 0.98599 | 0.97798 |
| samT | 0.97856 | 0.99145 | 0.97866 |
| shrinkT | 0.97847 | 0.98146 | 0.98823 |
| ibmT | 0.98711 | 0.98799 | 0.97885 |

**Dataset 19**

**1.** http://www.ncbi.nlm.nih.gov/sites/entrez?Db=Pubmed&term=15374838[UID]

**2.** GSE1650

**3.** 18 severe emphysema samples:

GSM28357, GSM28359, GSM28361, GSM28363-28369,

GSM28371, GSM28373, GSM28375, GSM28377, GSM28379,

GSM28381, GSM28383, GSM28385

**4.** 12 no/mild emphysema samples:

GSM28358, GSM28360, GSM28362, GSM28370, GSM28372,

GSM28374, GSM28376, GSM28378, GSM28380, GSM28382,

GSM28384, GSM28386

**5.** 8 probesets.

"211959_at", "204640_s_at", "219127_at", "201744_s_at", "202291_s_at",

"202995_s_at", "200872_at", "216652_s_at"

**6.** MAS5.0

**7.** RMA- and DFW-preprocessed data were obtained from a total of 30 samples in GSE1650.

**8-1.**

| MAS-preprocessed data | | |  |  |  |
| --- | --- | --- | --- | --- | --- |
| Method | 0% | 25% | 50% | 75% | 100% |
| *w* | 250.00 | 663.50 | 1036.00 | 3493.25 | 8825.00 |
| AD | 196.00 | 1491.50 | 2502.00 | 3592.25 | 5997.00 |
| WAD | 5.00 | 22.50 | 134.00 | 612.25 | 2006.00 |
| FC | 221.00 | 1305.75 | 2458.00 | 3453.25 | 5040.00 |
| RP | 425.00 | 2994.50 | 5679.00 | 8108.00 | 10575.00 |
| modT | 1.00 | 15.25 | 39.50 | 74.00 | 228.00 |
| samT | 1.00 | 22.25 | 61.50 | 110.75 | 319.00 |
| shrinkT | 1.00 | 35.75 | 116.50 | 207.75 | 776.00 |
| ibmT | 1.00 | 6.50 | 23.00 | 65.25 | 381.00 |
| RMA-preprocessed data | | |  |  |  |
| Method | 0% | 25% | 50% | 75% | 100% |
| *w* | 192.00 | 713.25 | 1084.50 | 5571.00 | 14880.00 |
| AD | 2.00 | 58.75 | 140.00 | 508.25 | 13468.00 |
| WAD | 1.00 | 23.75 | 49.00 | 718.50 | 13577.00 |
| FC | 1.00 | 66.50 | 150.00 | 510.00 | 12938.00 |
| RP | 2.00 | 87.00 | 170.50 | 697.00 | 17191.00 |
| modT | 2.00 | 42.00 | 88.00 | 931.25 | 11648.00 |
| samT | 1.00 | 32.25 | 61.00 | 802.25 | 11920.00 |
| shrinkT | 3.00 | 45.75 | 106.50 | 986.75 | 11541.00 |
| ibmT | 3.00 | 44.75 | 99.00 | 914.50 | 11720.00 |
| DFW-preprocessed data | | |  |  |  |
| Method | 0% | 25% | 50% | 75% | 100% |
| *w* | 275.00 | 970.25 | 1984.50 | 4621.50 | 17820.00 |
| AD | 44.00 | 130.50 | 311.50 | 1238.75 | 7260.00 |
| WAD | 49.00 | 97.00 | 268.50 | 1369.75 | 9206.00 |
| FC | 42.00 | 130.00 | 311.50 | 1238.75 | 7261.00 |
| RP | 7.00 | 69.25 | 133.50 | 955.75 | 8604.00 |
| modT | 1.00 | 94.00 | 140.00 | 258.00 | 6928.00 |
| samT | 1.00 | 73.00 | 100.50 | 206.50 | 6774.00 |
| shrinkT | 1.00 | 66.25 | 99.50 | 185.50 | 6145.00 |
| ibmT | 1.00 | 93.00 | 136.00 | 251.00 | 6828.00 |

**8-2.**

| Method | MAS | RMA | DFW |
| --- | --- | --- | --- |
| *w* | 0.88937 | 0.83071 | 0.80885 |
| AD | 0.88360 | 0.91297 | 0.93890 |
| WAD | 0.97625 | 0.91078 | 0.92676 |
| FC | 0.89260 | 0.91647 | 0.93892 |
| RP | 0.75371 | 0.88861 | 0.93642 |
| modT | 0.99718 | 0.91430 | 0.95507 |
| samT | 0.99591 | 0.91557 | 0.95732 |
| shrinkT | 0.99155 | 0.91363 | 0.96129 |
| ibmT | 0.99663 | 0.91392 | 0.95574 |

**Dataset 20**

**1.** http://www.ncbi.nlm.nih.gov/sites/entrez?Db=Pubmed&term=15598877[UID]

**2.** GSE1615

**3.** 4 untreated normal samples:

GSM27531-27534

**4.** 5 untreated PCOS samples:

GSM27536-27538, GSM27540, GSM27541

**5.** 8 probesets.

"205651_x_at", "202724_s_at", "218145_at", "203917_at", "205700_at",

"208740_at", "208741_at", "208742_s_at"

**6.** MAS5.0

**7.** RMA- and DFW-preprocessed data were obtained from a total of **13** samples in GSE1615.

**8-1.**

| MAS-preprocessed data | | |  |  |  |
| --- | --- | --- | --- | --- | --- |
| Method | 0% | 25% | 50% | 75% | 100% |
| *w* | 1061.00 | 5291.50 | 10331.50 | 11163.75 | 14505.00 |
| AD | 153.00 | 306.50 | 1675.50 | 3543.50 | 9837.00 |
| WAD | 29.00 | 185.25 | 1044.50 | 2209.25 | 6317.00 |
| FC | 149.00 | 528.25 | 1669.00 | 3412.50 | 9594.00 |
| RP | 202.00 | 346.25 | 1950.50 | 4091.00 | 11916.00 |
| modT | 123.00 | 343.00 | 626.00 | 2144.75 | 4219.00 |
| samT | 150.00 | 239.50 | 536.50 | 2165.25 | 5199.00 |
| shrinkT | 158.00 | 297.50 | 572.50 | 2103.50 | 4633.00 |
| ibmT | 106.00 | 292.75 | 632.50 | 1974.25 | 3624.00 |
| RMA-preprocessed data | | |  |  |  |
| Method | 0% | 25% | 50% | 75% | 100% |
| *w* | 888.00 | 5051.25 | 9858.50 | 11803.75 | 20209.00 |
| AD | 57.00 | 544.75 | 788.00 | 3594.50 | 16732.00 |
| WAD | 29.00 | 1422.00 | 2505.00 | 5105.75 | 14392.00 |
| FC | 102.00 | 457.00 | 878.50 | 3823.50 | 16772.00 |
| RP | 39.00 | 659.25 | 913.00 | 3792.00 | 14509.00 |
| modT | 280.00 | 479.75 | 2072.00 | 4646.50 | 12991.00 |
| samT | 281.00 | 425.00 | 1796.50 | 4294.25 | 12983.00 |
| shrinkT | 244.00 | 359.75 | 1763.50 | 4078.25 | 14194.00 |
| ibmT | 222.00 | 450.50 | 2414.00 | 4401.50 | 14666.00 |
| DFW-preprocessed data | | |  |  |  |
| Method | 0% | 25% | 50% | 75% | 100% |
| *w* | 727.00 | 6399.00 | 9475.50 | 12592.75 | 22119.00 |
| AD | 56.00 | 1421.75 | 3642.50 | 5640.75 | 19243.00 |
| WAD | 40.00 | 3228.75 | 4064.50 | 5271.50 | 17863.00 |
| FC | 56.00 | 1421.00 | 3643.50 | 5642.50 | 19242.00 |
| RP | 16.00 | 1712.75 | 2971.50 | 5461.50 | 15180.00 |
| modT | 438.00 | 1074.50 | 3387.50 | 5790.00 | 18720.00 |
| samT | 527.00 | 1242.50 | 3748.00 | 6286.25 | 18961.00 |
| shrinkT | 255.00 | 524.50 | 1785.50 | 3405.00 | 18775.00 |
| ibmT | 424.00 | 1074.00 | 3936.50 | 5847.25 | 19090.00 |

**8-2.**

| Method | MAS | RMA | DFW |
| --- | --- | --- | --- |
| *w* | 0.62539 | 0.58016 | 0.55853 |
| AD | 0.87002 | 0.85171 | 0.76410 |
| WAD | 0.91549 | 0.81569 | 0.75256 |
| FC | 0.87175 | 0.84747 | 0.76408 |
| RP | 0.84520 | 0.85905 | 0.79512 |
| modT | 0.93808 | 0.84332 | 0.77392 |
| samT | 0.93134 | 0.85242 | 0.76058 |
| shrinkT | 0.93618 | 0.84961 | 0.82357 |
| ibmT | 0.94395 | 0.83526 | 0.76503 |

**Dataset 21**

**1.** http://www.ncbi.nlm.nih.gov/sites/entrez?Db=Pubmed&term=15598877[UID]

**2.** GSE1615

**3.** 4 untreated normal samples:

GSM27531-27534

**4.** 4 VPA-treated normal samples:

GSM27543, GSM27546, GSM27548, GSM27549

**5.** 8 probesets.

"205651_x_at", "202724_s_at", "218145_at", "203917_at", "205700_at",

"208740_at", "208741_at", "208742_s_at"

**6.** MAS5.0

**7.** RMA- and DFW-preprocessed data were obtained from a total of **13** samples in GSE1615.

**8-1.**

| MAS-preprocessed data | | |  |  |  |
| --- | --- | --- | --- | --- | --- |
| Method | 0% | 25% | 50% | 75% | 100% |
| *w* | 927.00 | 5263.25 | 11533.00 | 11963.00 | 16487.00 |
| AD | 495.00 | 2386.50 | 3137.50 | 6467.25 | 12247.00 |
| WAD | 232.00 | 1103.50 | 2908.00 | 3773.50 | 12026.00 |
| FC | 770.00 | 2362.75 | 3204.00 | 7245.75 | 11217.00 |
| RP | 881.00 | 3242.50 | 5083.50 | 11093.50 | 18086.00 |
| modT | 447.00 | 2856.75 | 3330.50 | 5527.25 | 11039.00 |
| samT | 827.00 | 2083.25 | 2990.50 | 5121.25 | 11605.00 |
| shrinkT | 466.00 | 2947.50 | 3205.50 | 5409.25 | 11015.00 |
| ibmT | 309.00 | 2143.50 | 2534.00 | 5897.25 | 11498.00 |
| RMA-preprocessed data | | |  |  |  |
| Method | 0% | 25% | 50% | 75% | 100% |
| *w* | 859.00 | 5353.25 | 9890.00 | 12701.50 | 21694.00 |
| AD | 245.00 | 3081.25 | 4523.50 | 12401.25 | 14161.00 |
| WAD | 113.00 | 3043.00 | 4316.00 | 12708.00 | 19038.00 |
| FC | 286.00 | 3096.25 | 4473.50 | 12500.75 | 14571.00 |
| RP | 311.00 | 2358.25 | 3884.00 | 11295.25 | 13618.00 |
| modT | 2289.00 | 4787.00 | 6271.00 | 10936.50 | 17474.00 |
| samT | 2513.00 | 5977.50 | 7442.50 | 10792.25 | 18180.00 |
| shrinkT | 2285.00 | 4687.50 | 6193.50 | 10960.25 | 17419.00 |
| ibmT | 2160.00 | 4533.75 | 6733.50 | 9682.75 | 17256.00 |
| DFW-preprocessed data | | |  |  |  |
| Method | 0% | 25% | 50% | 75% | 100% |
| *w* | 726.00 | 6391.00 | 9473.00 | 12596.00 | 22119.00 |
| AD | 48.00 | 3913.50 | 5303.50 | 5680.75 | 14446.00 |
| WAD | 44.00 | 5011.25 | 5757.00 | 6483.50 | 13191.00 |
| FC | 47.00 | 3913.75 | 5303.50 | 5681.00 | 14447.00 |
| RP | 54.00 | 2481.75 | 4054.50 | 5485.75 | 15947.00 |
| modT | 1714.00 | 6505.75 | 7614.50 | 9308.75 | 18931.00 |
| samT | 1881.00 | 7069.50 | 8224.50 | 9866.25 | 19269.00 |
| shrinkT | 1132.00 | 4024.00 | 4880.50 | 7313.75 | 15607.00 |
| ibmT | 1622.00 | 6388.50 | 7484.50 | 10564.75 | 18936.00 |

**8-2.**

| Method | MAS | RMA | DFW |
| --- | --- | --- | --- |
| *w* | 0.59429 | 0.55825 | 0.55870 |
| AD | 0.78772 | 0.69446 | 0.75483 |
| WAD | 0.84233 | 0.65951 | 0.73447 |
| FC | 0.78630 | 0.69214 | 0.75481 |
| RP | 0.67416 | 0.72656 | 0.76835 |
| modT | 0.80226 | 0.64047 | 0.62029 |
| samT | 0.81585 | 0.61306 | 0.59896 |
| shrinkT | 0.80451 | 0.64220 | 0.70876 |
| ibmT | 0.81668 | 0.64589 | 0.61074 |

**Dataset 22**

**1.** http://www.ncbi.nlm.nih.gov/sites/entrez?Db=Pubmed&term=16089502[UID]

**2.** GSE2666

**3.** 4 bone marrow Rho^high^ samples:

GSM51401, GSM51403, GSM51405, GSM51407

**4.** 4 bone marrow Rho^low^ samples:

GSM51402, GSM51404, GSM51406, GSM51408

**5.** 5 probesets.

"209560_s_at", "209993_at", "206176_at", "204695_at", "202917_s_at"

**6.** MAS5.0

**7.** RMA- and DFW-preprocessed data were obtained from a total of **18** samples in GSE2666.

**8-1.**

| 8-1. Percentile ranks for true positives (TPs) | | | |  |  |
| --- | --- | --- | --- | --- | --- |
| MAS-preprocessed data | | |  |  |  |
| Method | 0% | 25% | 50% | 75% | 100% |
| *w* | 3250.00 | 3443.00 | 5693.00 | 10155.00 | 14005.00 |
| AD | 2.00 | 2248.00 | 2423.00 | 4326.00 | 12112.00 |
| WAD | 7.00 | 555.00 | 1292.00 | 1896.00 | 8277.00 |
| FC | 2.00 | 2363.00 | 2380.00 | 4299.00 | 11922.00 |
| RP | 3.00 | 2399.00 | 2635.00 | 4734.00 | 15446.00 |
| modT | 51.00 | 368.00 | 4195.00 | 4352.00 | 5567.00 |
| samT | 28.00 | 462.00 | 3981.00 | 4125.00 | 4807.00 |
| shrinkT | 46.00 | 379.00 | 4065.00 | 4921.00 | 5338.00 |
| ibmT | 30.00 | 580.00 | 3012.00 | 3583.00 | 3866.00 |
| RMA-preprocessed data | | |  |  |  |
| Method | 0% | 25% | 50% | 75% | 100% |
| *w* | 3658.00 | 3951.00 | 5919.00 | 6302.00 | 13179.00 |
| AD | 11.00 | 395.00 | 783.00 | 947.00 | 1741.00 |
| WAD | 71.00 | 243.00 | 928.00 | 1164.00 | 1333.00 |
| FC | 8.00 | 503.00 | 852.00 | 957.00 | 1788.00 |
| RP | 10.00 | 153.00 | 444.00 | 1130.00 | 1470.00 |
| modT | 157.00 | 477.00 | 1631.00 | 6086.00 | 7355.00 |
| samT | 247.00 | 636.00 | 2199.00 | 7596.00 | 8907.00 |
| shrinkT | 182.00 | 497.00 | 1747.00 | 6679.00 | 7967.00 |
| ibmT | 105.00 | 517.00 | 1832.00 | 5624.00 | 6945.00 |
| DFW-preprocessed data | | |  |  |  |
| Method | 0% | 25% | 50% | 75% | 100% |
| *w* | 3468.00 | 4256.00 | 5723.00 | 10085.00 | 20137.00 |
| AD | 14.00 | 60.00 | 641.00 | 783.00 | 1041.00 |
| WAD | 32.00 | 43.00 | 964.00 | 1030.00 | 1102.00 |
| FC | 14.00 | 60.00 | 641.00 | 784.00 | 1041.00 |
| RP | 17.00 | 49.00 | 621.00 | 699.00 | 924.00 |
| modT | 390.00 | 908.00 | 2328.00 | 8797.00 | 8878.00 |
| samT | 420.00 | 1008.00 | 2573.00 | 9276.00 | 9349.00 |
| shrinkT | 311.00 | 687.00 | 1665.00 | 5886.00 | 5946.00 |
| ibmT | 369.00 | 873.00 | 2347.00 | 8851.00 | 8939.00 |

**8-2.**

| Method | MAS | RMA | DFW |
| --- | --- | --- | --- |
| *w* | 0.67204 | 0.70380 | 0.60810 |
| AD | 0.81061 | 0.96533 | 0.97734 |
| WAD | 0.89216 | 0.96657 | 0.97167 |
| FC | 0.81191 | 0.96326 | 0.97733 |
| RP | 0.77375 | 0.97134 | 0.97940 |
| modT | 0.86967 | 0.85913 | 0.80891 |
| samT | 0.87981 | 0.82431 | 0.79701 |
| shrinkT | 0.86773 | 0.84687 | 0.87001 |
| ibmT | 0.90075 | 0.86527 | 0.80821 |

**Dataset 23**

**1.** http://www.ncbi.nlm.nih.gov/sites/entrez?Db=Pubmed&term=16089502[UID]

**2.** GSE2666

**3.** 5 Umbilical cord blood Rho^high^ samples:

GSM51391, GSM51393, GSM51395, GSM51397, GSM51399

**4.** 5 Umbilical cord blood Rho^low^ samples:

GSM51392, GSM51394, GSM51396, GSM51398, GSM51400

**5.** 6 probesets.

"209560_s_at", "209994_s_at", "206176_at", "204695_at", "202917_s_at",

"214370_at"

**6.** MAS5.0

**7.** RMA- and DFW-preprocessed data were obtained from a total of **18** samples in GSE2666.

**8-1.**

| 8-1. Percentile ranks for true positives (TPs) | | | |  |  |
| --- | --- | --- | --- | --- | --- |
| MAS-preprocessed data | | |  |  |  |
| Method | 0% | 25% | 50% | 75% | 100% |
| *w* | 2574.00 | 5180.75 | 9312.00 | 11440.00 | 17763.00 |
| AD | 12.00 | 312.75 | 2044.50 | 3210.75 | 4931.00 |
| WAD | 12.00 | 78.25 | 347.00 | 2031.00 | 3386.00 |
| FC | 8.00 | 405.50 | 1961.00 | 4683.50 | 6505.00 |
| RP | 14.00 | 328.50 | 1774.00 | 4282.25 | 6311.00 |
| modT | 30.00 | 125.00 | 913.50 | 2704.00 | 5163.00 |
| samT | 29.00 | 124.00 | 1004.00 | 2911.50 | 4883.00 |
| shrinkT | 29.00 | 126.25 | 926.50 | 2719.00 | 4955.00 |
| ibmT | 43.00 | 64.00 | 961.50 | 2357.75 | 3738.00 |
| RMA-preprocessed data | | |  |  |  |
| Method | 0% | 25% | 50% | 75% | 100% |
| *w* | 3181.00 | 4533.00 | 9068.50 | 12797.00 | 20405.00 |
| AD | 9.00 | 114.00 | 192.00 | 675.75 | 1263.00 |
| WAD | 26.00 | 60.50 | 698.50 | 2346.00 | 5709.00 |
| FC | 6.00 | 165.25 | 317.00 | 699.00 | 1346.00 |
| RP | 12.00 | 74.50 | 280.50 | 1025.00 | 1577.00 |
| modT | 143.00 | 318.00 | 547.00 | 1001.00 | 2721.00 |
| samT | 94.00 | 281.00 | 571.50 | 792.25 | 1849.00 |
| shrinkT | 180.00 | 358.50 | 604.00 | 1220.75 | 3160.00 |
| ibmT | 104.00 | 295.00 | 552.50 | 809.25 | 2395.00 |
| DFW-preprocessed data | | |  |  |  |
| Method | 0% | 25% | 50% | 75% | 100% |
| *w* | 3399.00 | 5733.50 | 7976.50 | 17479.50 | 21036.00 |
| AD | 10.00 | 158.75 | 802.00 | 1368.00 | 1541.00 |
| WAD | 20.00 | 187.00 | 1163.00 | 1722.00 | 3836.00 |
| FC | 9.00 | 161.25 | 805.00 | 1368.50 | 1541.00 |
| RP | 10.00 | 85.25 | 499.00 | 1328.25 | 1547.00 |
| modT | 232.00 | 517.75 | 1440.00 | 2881.25 | 3665.00 |
| samT | 248.00 | 546.50 | 1524.50 | 3042.50 | 3846.00 |
| shrinkT | 182.00 | 382.25 | 1010.00 | 1941.50 | 2473.00 |
| ibmT | 224.00 | 508.25 | 1441.50 | 2881.00 | 3651.00 |

**8-2.**

| Method | MAS | RMA | DFW |
| --- | --- | --- | --- |
| *w* | 0.58931 | 0.56036 | 0.50622 |
| AD | 0.90763 | 0.98085 | 0.96532 |
| WAD | 0.95023 | 0.92628 | 0.94066 |
| FC | 0.88110 | 0.97826 | 0.96527 |
| RP | 0.88915 | 0.97450 | 0.96943 |
| modT | 0.92397 | 0.96012 | 0.92273 |
| samT | 0.92311 | 0.96921 | 0.91852 |
| shrinkT | 0.92524 | 0.95339 | 0.94704 |
| ibmT | 0.93812 | 0.96493 | 0.92298 |

**Dataset 24**

**1.** http://www.ncbi.nlm.nih.gov/sites/entrez?Db=Pubmed&term=17251300[UID]

**2.** GSE6740

**3.** 10 (A + C) CD4+ cells:

GSM154936, GSM155180, GSM155182, GSM155184, GSM155186,

GSM155189, GSM155192, GSM155200, GSM155202, GSM155204

**4.** 10 (N + L) CD4+ cells:

GSM155218, GSM155220, GSM155222, GSM155224, GSM155226,

GSM155228, GSM155230, GSM155233, GSM155235, GSM155237

**5.** 40 probesets.

"201292_at", "201649_at", "201890_at", "202086_at", "202095_s_at",

"202145_at", "202270_at", "202446_s_at", "202589_at", "202869_at",

"203153_at", "203554_x_at", "204211_x_at", "204415_at", "204439_at",

"204747_at", "204972_at", "204994_at", "205241_at", "205483_s_at",

"205569_at", "205660_at", "205692_s_at", "206133_at", "206486_at",

"206513_at", "206991_s_at", "208965_s_at", "209417_s_at", "209969_s_at",

"213629_x_at", "213797_at", "214453_s_at", "217933_s_at", "218400_at",

"218543_s_at", "218741_at", "219352_at", "220169_at", "222154_s_at"

**6.** MAS5.0

**7.** RMA- and DFW-preprocessed data were obtained from a total of **40** samples in GSE6740.

**8-1.**

| 8-1. Percentile ranks for true positives (TPs) | | | |  |  |
| --- | --- | --- | --- | --- | --- |
| MAS-preprocessed data | | |  |  |  |
| Method | 0% | 25% | 50% | 75% | 100% |
| *w* | 1021.00 | 2791.00 | 4832.00 | 7416.50 | 15925.00 |
| AD | 1.00 | 67.75 | 205.00 | 552.00 | 3538.00 |
| WAD | 1.00 | 39.50 | 69.50 | 228.25 | 1608.00 |
| FC | 8.00 | 87.25 | 270.00 | 645.25 | 3507.00 |
| RP | 1.00 | 75.75 | 244.00 | 629.50 | 4854.00 |
| modT | 1.00 | 14.50 | 30.50 | 113.50 | 922.00 |
| samT | 1.00 | 15.50 | 51.50 | 91.00 | 1207.00 |
| shrinkT | 1.00 | 14.75 | 32.00 | 87.50 | 947.00 |
| ibmT | 1.00 | 12.75 | 29.50 | 105.75 | 800.00 |
| RMA-preprocessed data | | |  |  |  |
| Method | 0% | 25% | 50% | 75% | 100% |
| *w* | 1112.00 | 2936.75 | 4252.00 | 8101.50 | 18795.00 |
| AD | 1.00 | 24.00 | 63.00 | 173.50 | 921.00 |
| WAD | 1.00 | 27.50 | 81.00 | 391.75 | 5023.00 |
| FC | 5.00 | 42.25 | 88.00 | 197.75 | 894.00 |
| RP | 1.00 | 24.25 | 63.50 | 173.00 | 1142.00 |
| modT | 1.00 | 24.00 | 41.50 | 93.50 | 408.00 |
| samT | 1.00 | 24.00 | 41.00 | 95.25 | 421.00 |
| shrinkT | 1.00 | 19.75 | 34.50 | 87.25 | 344.00 |
| ibmT | 1.00 | 21.25 | 35.50 | 86.75 | 388.00 |
| DFW-preprocessed data | | |  |  |  |
| Method | 0% | 25% | 50% | 75% | 100% |
| *w* | 1248.00 | 4832.25 | 6312.00 | 11246.25 | 21273.00 |
| AD | 2.00 | 83.50 | 202.50 | 336.00 | 1932.00 |
| WAD | 4.00 | 84.25 | 206.00 | 391.25 | 2324.00 |
| FC | 9.00 | 82.50 | 202.50 | 335.75 | 1931.00 |
| RP | 1.00 | 31.50 | 93.50 | 215.00 | 1913.00 |
| modT | 1.00 | 22.50 | 43.00 | 132.75 | 3634.00 |
| samT | 1.00 | 20.50 | 42.50 | 130.50 | 3432.00 |
| shrinkT | 1.00 | 19.50 | 39.50 | 111.25 | 2023.00 |
| ibmT | 1.00 | 22.50 | 43.00 | 131.50 | 3616.00 |

**8-2.**

| Method | MAS | RMA | DFW |
| --- | --- | --- | --- |
| *w* | 0.74522 | 0.72354 | 0.63685 |
| AD | 0.97646 | 0.99412 | 0.98752 |
| WAD | 0.99274 | 0.97951 | 0.98508 |
| FC | 0.97531 | 0.99334 | 0.98751 |
| RP | 0.97049 | 0.99322 | 0.99195 |
| modT | 0.99632 | 0.99770 | 0.99293 |
| samT | 0.99624 | 0.99767 | 0.99336 |
| shrinkT | 0.99676 | 0.99802 | 0.99553 |
| ibmT | 0.99644 | 0.99782 | 0.99300 |

**Dataset 25**

**1.** http://www.ncbi.nlm.nih.gov/sites/entrez?Db=Pubmed&term=17251300[UID]

**2.** GSE6740

**3.** 10 (A + C) CD8+ cells:

GSM155179, GSM155181, GSM155183, GSM155185, GSM155187,

GSM155190, GSM155195, GSM155201, GSM155203, GSM155206

**4.** 10 (N + L) CD8+ cells:

GSM155219, GSM155221, GSM155223, GSM155225, GSM155227,

GSM155229, GSM155232, GSM155234, GSM155236, GSM155238

**5.** 62 probesets.

"201292_at", "201649_at", "201890_at", "202086_at", "202095_s_at",

"202145_at", "202270_at", "202446_s_at", "202589_at", "202869_at",

"203153_at", "203554_x_at", "204211_x_at", "204415_at", "204439_at",

"204747_at", "204972_at", "204994_at", "205241_at", "205483_s_at",

"205569_at", "205660_at", "205692_s_at", "206133_at", "206486_at",

"206513_at", "206991_s_at", "208965_s_at", "209417_s_at", "209969_s_at",

"213629_x_at", "213797_at", "214453_s_at", "217933_s_at", "218400_at",

"218543_s_at", "218741_at", "219352_at", "220169_at", "222154_s_at",

"201930_at", "202760_s_at", "203236_s_at", "203658_at", "204070_at",

"204205_at", "205098_at", "205898_at", "206632_s_at", "212048_s_at",

"213060_s_at", "214617_at", "215691_x_at", "35254_at", "200731_s_at",

"209741_x_at", "212762_s_at", "212870_at", "213222_at", "218486_at",

"219526_at", "221763_at"

**6.** MAS5.0

**7.** RMA- and DFW-preprocessed data were obtained from a total of **40** samples in GSE6740.

**8-1.**

| 8-1. Percentile ranks for true positives (TPs) | | | |  |  |
| --- | --- | --- | --- | --- | --- |
| MAS-preprocessed data | | |  |  |  |
| Method | 0% | 25% | 50% | 75% | 100% |
| *w* | 340.00 | 2271.25 | 4023.50 | 7191.00 | 13797.00 |
| AD | 1.00 | 184.50 | 438.50 | 1352.00 | 4219.00 |
| WAD | 1.00 | 67.25 | 178.50 | 538.50 | 3021.00 |
| FC | 2.00 | 160.25 | 425.00 | 1405.50 | 4311.00 |
| RP | 1.00 | 190.00 | 534.00 | 1944.00 | 7236.00 |
| modT | 1.00 | 35.25 | 97.00 | 235.00 | 1131.00 |
| samT | 1.00 | 30.50 | 134.00 | 339.50 | 1583.00 |
| shrinkT | 1.00 | 34.25 | 102.00 | 240.75 | 1212.00 |
| ibmT | 1.00 | 35.75 | 86.00 | 226.75 | 1020.00 |
| RMA-preprocessed data | | |  |  |  |
| Method | 0% | 25% | 50% | 75% | 100% |
| *w* | 272.00 | 2320.75 | 4290.50 | 8050.25 | 20361.00 |
| AD | 2.00 | 50.25 | 144.50 | 400.25 | 2943.00 |
| WAD | 3.00 | 72.75 | 222.50 | 629.00 | 8682.00 |
| FC | 4.00 | 59.25 | 144.50 | 448.00 | 2816.00 |
| RP | 2.00 | 48.25 | 135.00 | 381.25 | 3307.00 |
| modT | 1.00 | 78.50 | 215.50 | 574.75 | 3949.00 |
| samT | 1.00 | 65.25 | 206.00 | 543.75 | 3907.00 |
| shrinkT | 1.00 | 53.75 | 186.00 | 501.25 | 3547.00 |
| ibmT | 1.00 | 59.25 | 200.50 | 508.75 | 3947.00 |
| DFW-preprocessed data | | |  |  |  |
| Method | 0% | 25% | 50% | 75% | 100% |
| *w* | 672.00 | 3997.00 | 6409.50 | 11804.75 | 21253.00 |
| AD | 2.00 | 140.25 | 349.50 | 840.50 | 5903.00 |
| WAD | 4.00 | 139.50 | 377.50 | 690.75 | 6659.00 |
| FC | 9.00 | 138.75 | 349.50 | 841.00 | 5902.00 |
| RP | 1.00 | 64.50 | 222.50 | 562.25 | 6151.00 |
| modT | 1.00 | 67.25 | 253.50 | 760.00 | 3875.00 |
| samT | 1.00 | 63.25 | 239.50 | 730.50 | 3731.00 |
| shrinkT | 1.00 | 55.25 | 202.00 | 578.75 | 2851.00 |
| ibmT | 1.00 | 65.75 | 245.50 | 739.25 | 3850.00 |

**8-2.**

| Method | MAS | RMA | DFW |
| --- | --- | --- | --- |
| *w* | 0.76932 | 0.72767 | 0.62845 |
| AD | 0.96430 | 0.98355 | 0.97050 |
| WAD | 0.98463 | 0.96410 | 0.96459 |
| FC | 0.96503 | 0.98357 | 0.97048 |
| RP | 0.95021 | 0.98436 | 0.97696 |
| modT | 0.99304 | 0.97976 | 0.97544 |
| samT | 0.99027 | 0.98060 | 0.97633 |
| shrinkT | 0.99313 | 0.98248 | 0.98081 |
| ibmT | 0.99330 | 0.98135 | 0.97578 |

**Dataset 26**

**1.** http://www.ncbi.nlm.nih.gov/sites/entrez?Db=Pubmed&term=18058819[UID]

**2.** GSE9574

**3.** 15 reduction mammoplasty patient samples:

GSM241999-242013

**4.** 14 breast cancer patient samples:

GSM242014-242027

**5.** 5 probesets.

"209774_x_at", "209189_at", "202768_at", "208960_s_at", "208961_s_at"

**6.** MAS5.0

**7.** RMA- and DFW-preprocessed data were obtained from a total of 29 samples in GSE9574.

**8-1.**

| 8-1. Percentile ranks for true positives (TPs) | | | |  |  |
| --- | --- | --- | --- | --- | --- |
| MAS-preprocessed data | | |  |  |  |
| Method | 0% | 25% | 50% | 75% | 100% |
| *w* | 1156.00 | 3203.00 | 3587.00 | 5531.00 | 15025.00 |
| AD | 1.00 | 2.00 | 5.00 | 22.00 | 31.00 |
| WAD | 1.00 | 2.00 | 6.00 | 16.00 | 24.00 |
| FC | 1.00 | 3.00 | 29.00 | 69.00 | 126.00 |
| RP | 1.00 | 2.00 | 3.00 | 39.00 | 54.00 |
| modT | 4.00 | 9.00 | 43.00 | 46.00 | 85.00 |
| samT | 1.00 | 4.00 | 21.00 | 30.00 | 52.00 |
| shrinkT | 2.00 | 7.00 | 36.00 | 39.00 | 73.00 |
| ibmT | 3.00 | 7.00 | 35.00 | 41.00 | 64.00 |
| RMA-preprocessed data | | |  |  |  |
| Method | 0% | 25% | 50% | 75% | 100% |
| *w* | 1135.00 | 2105.00 | 2796.00 | 4182.00 | 10009.00 |
| AD | 1.00 | 2.00 | 8.00 | 22.00 | 87.00 |
| WAD | 2.00 | 6.00 | 8.00 | 27.00 | 414.00 |
| FC | 1.00 | 2.00 | 23.00 | 42.00 | 128.00 |
| RP | 1.00 | 2.00 | 9.00 | 23.00 | 106.00 |
| modT | 2.00 | 7.00 | 43.00 | 62.00 | 154.00 |
| samT | 2.00 | 4.00 | 34.00 | 44.00 | 116.00 |
| shrinkT | 2.00 | 7.00 | 43.00 | 61.00 | 146.00 |
| ibmT | 2.00 | 7.00 | 39.00 | 58.00 | 128.00 |
| DFW-preprocessed data | | |  |  |  |
| Method | 0% | 25% | 50% | 75% | 100% |
| *w* | 3545.00 | 3982.00 | 4062.00 | 4327.00 | 12765.00 |
| AD | 2.00 | 5.00 | 7.00 | 62.00 | 1614.00 |
| WAD | 4.00 | 8.00 | 13.00 | 70.00 | 2547.00 |
| FC | 2.00 | 6.00 | 8.00 | 63.00 | 1612.00 |
| RP | 1.00 | 4.00 | 5.00 | 39.00 | 1025.00 |
| modT | 2.00 | 6.00 | 69.00 | 98.00 | 144.00 |
| samT | 2.00 | 6.00 | 67.00 | 98.00 | 139.00 |
| shrinkT | 2.00 | 6.00 | 60.00 | 86.00 | 122.00 |
| ibmT | 2.00 | 6.00 | 67.00 | 99.00 | 146.00 |

**8-2.**

| Method | MAS | RMA | DFW |
| --- | --- | --- | --- |
| *w* | 0.74426 | 0.81855 | 0.74265 |
| AD | 0.99959 | 0.99906 | 0.98496 |
| WAD | 0.99969 | 0.99603 | 0.97642 |
| FC | 0.99809 | 0.99838 | 0.98495 |
| RP | 0.99925 | 0.99887 | 0.99049 |
| modT | 0.99846 | 0.99773 | 0.99727 |
| samT | 0.99917 | 0.99834 | 0.99733 |
| shrinkT | 0.99873 | 0.99781 | 0.99766 |
| ibmT | 0.99879 | 0.99803 | 0.99726 |

**Dataset 27**

**1.** http://www.ncbi.nlm.nih.gov/sites/entrez?Db=Pubmed&term=16690749[UID]

**2.** GSE4917

**3.** 3 Dex-treated samples:

GSM109211, GSM109219, GSM109227

**4.** 3 Control (ethanol)-treated samples:

GSM109210, GSM109218, GSM109226

**5.** 5 probesets.

"210095_s_at", "212143_s_at", "205479_s_at", "211668_s_at", "204292_x_at"

**6.** RMA

**7.** DFW-preprocessed data were obtained from a total of 24 samples in GSE4917.

**8-1.**

| 8-1. Percentile ranks for true positives (TPs) | | | |  |  |
| --- | --- | --- | --- | --- | --- |
| MAS-preprocessed data | | |  |  |  |
| Method | 0% | 25% | 50% | 75% | 100% |
| *w* | 2976.00 | 5756.00 | 7697.00 | 8659.00 | 16864.00 |
| AD | 115.00 | 180.00 | 216.00 | 310.00 | 22105.00 |
| WAD | 39.00 | 55.00 | 86.00 | 93.00 | 22152.00 |
| FC | 65.00 | 175.00 | 227.00 | 315.00 | 19919.00 |
| RP | 177.00 | 271.00 | 339.00 | 488.00 | 18704.00 |
| modT | 13.00 | 99.00 | 313.00 | 1176.00 | 22104.00 |
| samT | 15.00 | 120.00 | 376.00 | 1294.00 | 22116.00 |
| shrinkT | 12.00 | 109.00 | 377.00 | 1505.00 | 22112.00 |
| ibmT | 5.00 | 50.00 | 99.00 | 459.00 | 22165.00 |
| RMA-preprocessed data | | |  |  |  |
| Method | 0% | 25% | 50% | 75% | 100% |
| *w* | 2276.00 | 3882.00 | 6517.00 | 6969.00 | 7881.00 |
| AD | 23.00 | 74.00 | 78.00 | 130.00 | 360.00 |
| WAD | 42.00 | 54.00 | 112.00 | 136.00 | 411.00 |
| FC | 33.00 | 59.00 | 68.00 | 103.00 | 360.00 |
| RP | 15.00 | 41.00 | 120.00 | 216.00 | 253.00 |
| modT | 31.00 | 42.00 | 282.00 | 1216.00 | 2891.00 |
| samT | 35.00 | 48.00 | 361.00 | 1233.00 | 2832.00 |
| shrinkT | 44.00 | 60.00 | 436.00 | 2015.00 | 4160.00 |
| ibmT | 27.00 | 36.00 | 253.00 | 859.00 | 2288.00 |
| DFW-preprocessed data | | |  |  |  |
| Method | 0% | 25% | 50% | 75% | 100% |
| *w* | 3358.00 | 5919.00 | 6455.00 | 11164.00 | 11815.00 |
| AD | 61.00 | 126.00 | 158.00 | 248.00 | 2266.00 |
| WAD | 52.00 | 160.00 | 192.00 | 264.00 | 2522.00 |
| FC | 61.00 | 124.00 | 158.00 | 249.00 | 2266.00 |
| RP | 52.00 | 59.00 | 92.00 | 191.00 | 1006.00 |
| modT | 201.00 | 219.00 | 1463.00 | 2646.00 | 4843.00 |
| samT | 258.00 | 280.00 | 1824.00 | 3223.00 | 5766.00 |
| shrinkT | 160.00 | 167.00 | 1138.00 | 2038.00 | 3810.00 |
| ibmT | 150.00 | 166.00 | 1336.00 | 2499.00 | 4790.00 |

**8-2.**

| Method | MAS | RMA | DFW |
| --- | --- | --- | --- |
| *w* | 0.62351 | 0.75303 | 0.65261 |
| AD | 0.79432 | 0.99416 | 0.97447 |
| WAD | 0.79881 | 0.99336 | 0.97150 |
| FC | 0.81429 | 0.99454 | 0.97448 |
| RP | 0.82077 | 0.99434 | 0.98757 |
| modT | 0.78732 | 0.96008 | 0.91600 |
| samT | 0.78538 | 0.95966 | 0.89823 |
| shrinkT | 0.78364 | 0.93985 | 0.93448 |
| ibmT | 0.79565 | 0.96905 | 0.91987 |

**Dataset 28**

**1.** http://www.ncbi.nlm.nih.gov/sites/entrez?Db=Pubmed&term=17854483[UID]

**2.** GSE7148

**3.** 7 high-lonely samples:

GSM172173-172179

**4.** 7 low-lonely samples:

GSM172180-172186

**5.** 10 probesets.

"202859_x_at", "211506_s_at", "201853_s_at", "39402_at", "205067_at",

"201694_s_at", "201693_s_at", "202768_at", "204748_at", "204415_at"

**6.** RMA

**7.** DFW-preprocessed data were obtained from a total of 14 samples in GSE7148.

**8-1.**

| 8-1. Percentile ranks for true positives (TPs) | | | |  |  |
| --- | --- | --- | --- | --- | --- |
| MAS-preprocessed data | | |  |  |  |
| Method | 0% | 25% | 50% | 75% | 100% |
| *w* | 1538.00 | 5565.25 | 7833.00 | 10716.25 | 15947.00 |
| AD | 36.00 | 431.00 | 2619.00 | 4143.50 | 8334.00 |
| WAD | 9.00 | 62.75 | 725.00 | 1642.00 | 3335.00 |
| FC | 31.00 | 48.75 | 246.00 | 2154.00 | 7218.00 |
| RP | 29.00 | 321.50 | 1934.00 | 6299.75 | 11279.00 |
| modT | 764.00 | 2216.75 | 3439.50 | 5212.00 | 12422.00 |
| samT | 738.00 | 2203.25 | 3601.00 | 5303.75 | 12311.00 |
| shrinkT | 651.00 | 1953.25 | 3307.50 | 4877.75 | 11868.00 |
| ibmT | 523.00 | 1554.25 | 2516.50 | 3965.50 | 11287.00 |
| RMA-preprocessed data | | |  |  |  |
| Method | 0% | 25% | 50% | 75% | 100% |
| *w* | 1320.00 | 5170.00 | 9199.00 | 12119.25 | 13746.00 |
| AD | 8.00 | 28.75 | 108.00 | 200.50 | 958.00 |
| WAD | 26.00 | 99.75 | 293.50 | 449.75 | 912.00 |
| FC | 4.00 | 10.25 | 37.00 | 109.00 | 748.00 |
| RP | 10.00 | 50.50 | 140.50 | 366.75 | 905.00 |
| modT | 573.00 | 2293.25 | 3971.50 | 4584.50 | 8557.00 |
| samT | 222.00 | 1021.00 | 2430.00 | 2930.50 | 6051.00 |
| shrinkT | 584.00 | 2327.75 | 4025.50 | 4635.50 | 8619.00 |
| ibmT | 485.00 | 2023.00 | 3754.50 | 4292.50 | 8129.00 |
| DFW-preprocessed data | | |  |  |  |
| Method | 0% | 25% | 50% | 75% | 100% |
| *w* | 1739.00 | 7572.75 | 9708.50 | 13344.25 | 18844.00 |
| AD | 3.00 | 50.25 | 143.00 | 503.00 | 789.00 |
| WAD | 15.00 | 99.00 | 221.50 | 462.50 | 1193.00 |
| FC | 3.00 | 50.75 | 144.00 | 501.75 | 789.00 |
| RP | 10.00 | 66.50 | 194.50 | 433.25 | 1051.00 |
| modT | 935.00 | 3185.25 | 3221.50 | 5712.25 | 7471.00 |
| samT | 989.00 | 3312.75 | 3341.00 | 5862.50 | 7644.00 |
| shrinkT | 523.00 | 2037.00 | 2056.50 | 4013.75 | 5551.00 |
| ibmT | 920.00 | 3124.00 | 3155.50 | 5618.50 | 7355.00 |

**8-2.**

| Method | MAS | RMA | DFW |
| --- | --- | --- | --- |
| *w* | 0.62270 | 0.61776 | 0.51926 |
| AD | 0.87720 | 0.99200 | 0.98812 |
| WAD | 0.95566 | 0.98508 | 0.98270 |
| FC | 0.93358 | 0.99488 | 0.98810 |
| RP | 0.83960 | 0.98946 | 0.98705 |
| modT | 0.78696 | 0.83202 | 0.81788 |
| samT | 0.78584 | 0.89826 | 0.81213 |
| shrinkT | 0.79939 | 0.83009 | 0.87514 |
| ibmT | 0.82802 | 0.84363 | 0.82096 |

**Dataset 29**

**1.** http://www.ncbi.nlm.nih.gov/sites/entrez?Db=Pubmed&term=15583081[UID]

**2.** GSE5967

**3.** 7 patient samples:

GSM138597-138603

**4.** 7 control samples:

GSM138611-138617

**5.** 6 probesets.

"211862_x_at", "210564_x_at", "208485_x_at", "211317_s_at", "214486_x_at",

"221477_s_at"

**6.** RMA

**7.** DFW-preprocessed data were obtained from a total of **21** samples in GSE5967.

**8-1.**

| 8-1. Percentile ranks for true positives (TPs) | | | |  |  |
| --- | --- | --- | --- | --- | --- |
| MAS-preprocessed data | | |  |  |  |
| Method | 0% | 25% | 50% | 75% | 100% |
| *w* | 617.00 | 646.50 | 997.50 | 1344.75 | 2355.00 |
| AD | 123.00 | 902.00 | 1066.50 | 1774.00 | 2260.00 |
| WAD | 2.00 | 37.25 | 76.50 | 251.50 | 530.00 |
| FC | 247.00 | 743.75 | 841.00 | 1437.75 | 1635.00 |
| RP | 233.00 | 1816.00 | 2079.50 | 3379.50 | 4283.00 |
| modT | 38.00 | 59.75 | 145.00 | 226.50 | 258.00 |
| samT | 32.00 | 77.00 | 178.00 | 301.50 | 335.00 |
| shrinkT | 32.00 | 65.25 | 147.00 | 251.25 | 279.00 |
| ibmT | 16.00 | 36.50 | 82.50 | 115.75 | 140.00 |
| RMA-preprocessed data | | |  |  |  |
| Method | 0% | 25% | 50% | 75% | 100% |
| *w* | 567.00 | 762.25 | 1078.00 | 1453.75 | 1877.00 |
| AD | 23.00 | 27.50 | 41.50 | 80.25 | 150.00 |
| WAD | 15.00 | 19.00 | 50.50 | 124.00 | 160.00 |
| FC | 30.00 | 38.25 | 56.50 | 83.00 | 150.00 |
| RP | 12.00 | 15.75 | 32.00 | 53.50 | 139.00 |
| modT | 10.00 | 20.00 | 39.00 | 47.50 | 79.00 |
| samT | 3.00 | 12.50 | 18.00 | 28.75 | 56.00 |
| shrinkT | 11.00 | 22.75 | 41.00 | 49.50 | 82.00 |
| ibmT | 11.00 | 21.75 | 41.00 | 47.50 | 88.00 |
| DFW-preprocessed data | | |  |  |  |
| Method | 0% | 25% | 50% | 75% | 100% |
| *w* | 762.00 | 824.75 | 1056.00 | 1782.25 | 2608.00 |
| AD | 127.00 | 232.25 | 262.50 | 406.00 | 841.00 |
| WAD | 118.00 | 223.75 | 253.00 | 392.50 | 821.00 |
| FC | 127.00 | 233.00 | 262.00 | 405.75 | 842.00 |
| RP | 23.00 | 30.50 | 34.50 | 114.25 | 285.00 |
| modT | 41.00 | 50.00 | 104.50 | 160.50 | 224.00 |
| samT | 42.00 | 50.50 | 105.50 | 163.50 | 222.00 |
| shrinkT | 36.00 | 42.50 | 80.00 | 118.25 | 149.00 |
| ibmT | 39.00 | 49.00 | 103.00 | 162.25 | 221.00 |

**8-2.**

| Method | MAS | RMA | DFW |
| --- | --- | --- | --- |
| *w* | 0.94811 | 0.94901 | 0.93840 |
| AD | 0.94500 | 0.99737 | 0.98393 |
| WAD | 0.99253 | 0.99692 | 0.98446 |
| FC | 0.95593 | 0.99704 | 0.98392 |
| RP | 0.89380 | 0.99802 | 0.99606 |
| modT | 0.99364 | 0.99843 | 0.99503 |
| samT | 0.99186 | 0.99912 | 0.99499 |
| shrinkT | 0.99321 | 0.99833 | 0.99637 |
| ibmT | 0.99665 | 0.99832 | 0.99508 |

**Dataset 30**

**1.** http://www.ncbi.nlm.nih.gov/sites/entrez?Db=Pubmed&term=17264171[UID]

**2.** GSE6011

**3.** 14 normal skeletal muscle samples:

GSM139501-139514

**4.** 23 DMD skeletal muscle samples:

GSM139515-139537

**5.** 10 probesets.

"202310_s_at", "211161_s_at", "205132_at", "205940_at", "206717_at",

"202965_s_at", "206633_at", "221355_at", "204948_s_at", "208782_at"

**6.** RMA

**7.** DFW-preprocessed data were obtained from a total of 37 samples in GSE6011.

**8-1.**

| 8-1. Percentile ranks for true positives (TPs) | | | |  |  |
| --- | --- | --- | --- | --- | --- |
| MAS-preprocessed data | | |  |  |  |
| Method | 0% | 25% | 50% | 75% | 100% |
| *w* | 213.00 | 498.75 | 1659.00 | 3868.25 | 11242.00 |
| AD | 1.00 | 15.00 | 80.00 | 448.25 | 19899.00 |
| WAD | 1.00 | 4.75 | 24.50 | 170.00 | 19598.00 |
| FC | 1.00 | 29.00 | 151.50 | 545.50 | 20231.00 |
| RP | 1.00 | 13.25 | 104.50 | 681.25 | 14867.00 |
| modT | 2.00 | 24.75 | 121.00 | 327.00 | 20556.00 |
| samT | 2.00 | 18.00 | 82.00 | 246.75 | 20335.00 |
| shrinkT | 2.00 | 22.50 | 106.00 | 300.25 | 20533.00 |
| ibmT | 2.00 | 23.00 | 116.50 | 311.25 | 20523.00 |
| RMA-preprocessed data | | |  |  |  |
| Method | 0% | 25% | 50% | 75% | 100% |
| *w* | 189.00 | 530.75 | 2077.50 | 2607.00 | 6542.00 |
| AD | 1.00 | 5.50 | 20.00 | 128.25 | 2492.00 |
| WAD | 2.00 | 4.25 | 34.50 | 121.25 | 2683.00 |
| FC | 1.00 | 7.75 | 25.00 | 182.00 | 2465.00 |
| RP | 1.00 | 6.25 | 20.50 | 166.75 | 2666.00 |
| modT | 2.00 | 17.75 | 90.50 | 239.25 | 2524.00 |
| samT | 2.00 | 16.50 | 70.00 | 218.00 | 2360.00 |
| shrinkT | 2.00 | 17.75 | 88.00 | 237.75 | 2497.00 |
| ibmT | 2.00 | 17.25 | 79.50 | 234.50 | 2569.00 |
| DFW-preprocessed data | | |  |  |  |
| Method | 0% | 25% | 50% | 75% | 100% |
| *w* | 457.00 | 1381.25 | 4908.00 | 10103.00 | 11423.00 |
| AD | 1.00 | 6.75 | 36.00 | 240.50 | 6461.00 |
| WAD | 1.00 | 6.50 | 44.50 | 252.00 | 6680.00 |
| FC | 1.00 | 6.50 | 36.50 | 241.25 | 6464.00 |
| RP | 1.00 | 4.25 | 18.00 | 228.75 | 5986.00 |
| modT | 2.00 | 21.25 | 108.00 | 249.25 | 2492.00 |
| samT | 2.00 | 20.75 | 106.50 | 242.75 | 2351.00 |
| shrinkT | 2.00 | 21.00 | 108.00 | 245.25 | 2297.00 |
| ibmT | 2.00 | 21.00 | 107.00 | 248.25 | 2459.00 |

**8-2.**

| Method | MAS | RMA | DFW |
| --- | --- | --- | --- |
| *w* | 0.87663 | 0.90274 | 0.74906 |
| AD | 0.85386 | 0.97978 | 0.95834 |
| WAD | 0.87303 | 0.98054 | 0.95814 |
| FC | 0.85138 | 0.97946 | 0.95831 |
| RP | 0.86495 | 0.97889 | 0.96121 |
| modT | 0.86769 | 0.98117 | 0.97897 |
| samT | 0.86288 | 0.98189 | 0.97950 |
| shrinkT | 0.86740 | 0.98128 | 0.97989 |
| ibmT | 0.87021 | 0.98087 | 0.97922 |

**Dataset 31**

**1.** http://www.ncbi.nlm.nih.gov/sites/entrez?Db=Pubmed&term=17660348[UID]

**2.** GSE8562

**3.** 3 MCF7/c samples:

GSM212605-212607

**4.** 3 MCF7/XBP1 samples:

GSM212608-212610

**5.** 8 probesets.

"213419_at", "202224_at", "206569_at", "202431_s_at", "209803_s_at",

"217728_at", "208900_s_at", "200792_at"

**6.** RMA

**7.** DFW-preprocessed data were obtained from a total of 6 samples in GSE8562.

**8-1.**

| 8-1. Percentile ranks for true positives (TPs) | | | |  |  |
| --- | --- | --- | --- | --- | --- |
| MAS-preprocessed data | | |  |  |  |
| Method | 0% | 25% | 50% | 75% | 100% |
| *w* | 965.00 | 1465.75 | 2479.00 | 6742.75 | 12159.00 |
| AD | 322.00 | 860.75 | 2501.00 | 4899.25 | 7500.00 |
| WAD | 31.00 | 140.25 | 212.50 | 984.25 | 2415.00 |
| FC | 292.00 | 791.25 | 2277.50 | 4658.50 | 6590.00 |
| RP | 393.00 | 878.00 | 2848.50 | 5393.75 | 7954.00 |
| modT | 144.00 | 199.75 | 966.50 | 1672.25 | 3566.00 |
| samT | 90.00 | 217.75 | 1011.00 | 1737.50 | 3765.00 |
| shrinkT | 117.00 | 213.25 | 925.00 | 1793.50 | 3453.00 |
| ibmT | 30.00 | 67.75 | 287.00 | 886.25 | 1715.00 |
| RMA-preprocessed data | | |  |  |  |
| Method | 0% | 25% | 50% | 75% | 100% |
| *w* | 809.00 | 1639.25 | 3347.50 | 6759.75 | 13493.00 |
| AD | 62.00 | 137.50 | 319.50 | 483.00 | 541.00 |
| WAD | 24.00 | 151.25 | 293.00 | 385.25 | 2109.00 |
| FC | 54.00 | 135.00 | 339.50 | 522.25 | 542.00 |
| RP | 60.00 | 118.00 | 245.00 | 454.50 | 586.00 |
| modT | 35.00 | 123.00 | 179.50 | 493.50 | 1645.00 |
| samT | 39.00 | 176.75 | 248.00 | 673.50 | 2130.00 |
| shrinkT | 35.00 | 181.75 | 250.50 | 685.75 | 2300.00 |
| ibmT | 43.00 | 89.75 | 174.00 | 437.25 | 1382.00 |
| DFW-preprocessed data | | |  |  |  |
| Method | 0% | 25% | 50% | 75% | 100% |
| *w* | 771.00 | 1383.25 | 3142.50 | 8345.25 | 15347.00 |
| AD | 45.00 | 273.75 | 440.50 | 534.50 | 2415.00 |
| WAD | 27.00 | 229.75 | 427.00 | 775.25 | 3625.00 |
| FC | 45.00 | 273.75 | 440.50 | 535.50 | 2416.00 |
| RP | 39.00 | 226.00 | 294.50 | 320.25 | 1378.00 |
| modT | 5.00 | 216.00 | 550.50 | 1325.25 | 2273.00 |
| samT | 5.00 | 263.50 | 651.00 | 1573.50 | 2652.00 |
| shrinkT | 4.00 | 172.00 | 432.50 | 1043.00 | 1747.00 |
| ibmT | 3.00 | 207.75 | 507.50 | 1255.75 | 2187.00 |

**8-2.**

| Method | MAS | RMA | DFW |
| --- | --- | --- | --- |
| *w* | 0.79680 | 0.77628 | 0.74374 |
| AD | 0.86446 | 0.98650 | 0.97310 |
| WAD | 0.96846 | 0.97814 | 0.96279 |
| FC | 0.87411 | 0.98594 | 0.97308 |
| RP | 0.85113 | 0.98725 | 0.98343 |
| modT | 0.94122 | 0.98203 | 0.96384 |
| samT | 0.93804 | 0.97618 | 0.95728 |
| shrinkT | 0.94133 | 0.97484 | 0.97180 |
| ibmT | 0.97353 | 0.98384 | 0.96567 |

**Dataset 32**

**1.** http://www.ncbi.nlm.nih.gov/sites/entrez?Db=Pubmed&term=16620959[UID]

**2.** GSE1937

**3.** 4 TCPS samples:

GSM34736-34739

**4.** 4 DCOL samples:

GSM34744-34747

**5.** 12 probesets.

"210512_s_at", "210513_s_at", "211527_x_at", "212171_x_at", "209960_at",

"210755_at", "210998_s_at", "203665_at", "201147_s_at", "201148_s_at",

"201149_s_at", "201150_s_at"

**6.** RMA

**7.** DFW-preprocessed data were obtained from a total of **12** samples in GSE1937.

**8-1.**

| 8-1. Percentile ranks for true positives (TPs) | | | |  |  |
| --- | --- | --- | --- | --- | --- |
| MAS-preprocessed data | | |  |  |  |
| Method | 0% | 25% | 50% | 75% | 100% |
| *w* | 753.00 | 1472.50 | 1977.00 | 5069.00 | 6501.00 |
| AD | 3.00 | 1473.00 | 2954.00 | 4764.75 | 10489.00 |
| WAD | 1.00 | 194.25 | 674.00 | 1619.75 | 7359.00 |
| FC | 3.00 | 1378.25 | 2926.50 | 4557.25 | 10224.00 |
| RP | 14.00 | 700.50 | 2908.00 | 7827.50 | 15715.00 |
| modT | 1.00 | 244.75 | 1310.00 | 2277.25 | 7582.00 |
| samT | 2.00 | 528.75 | 1969.50 | 3219.75 | 9526.00 |
| shrinkT | 1.00 | 254.00 | 1301.50 | 2154.25 | 7563.00 |
| ibmT | 1.00 | 152.50 | 794.00 | 1505.25 | 6117.00 |
| RMA-preprocessed data | | |  |  |  |
| Method | 0% | 25% | 50% | 75% | 100% |
| *w* | 777.00 | 1231.00 | 1621.00 | 4500.25 | 8861.00 |
| AD | 2.00 | 367.25 | 864.00 | 2994.25 | 6298.00 |
| WAD | 1.00 | 159.25 | 496.50 | 1515.50 | 5640.00 |
| FC | 2.00 | 363.00 | 913.50 | 2984.25 | 6657.00 |
| RP | 1.00 | 236.25 | 739.00 | 2046.75 | 4929.00 |
| modT | 15.00 | 235.75 | 1820.00 | 3870.75 | 9029.00 |
| samT | 20.00 | 207.50 | 2064.00 | 4781.25 | 10224.00 |
| shrinkT | 24.00 | 196.75 | 2153.50 | 5419.25 | 10649.00 |
| ibmT | 12.00 | 189.25 | 1722.00 | 3699.50 | 9016.00 |
| DFW-preprocessed data | | |  |  |  |
| Method | 0% | 25% | 50% | 75% | 100% |
| *w* | 1059.00 | 1717.25 | 3055.00 | 5978.00 | 11549.00 |
| AD | 1.00 | 252.75 | 452.00 | 1679.00 | 5441.00 |
| WAD | 1.00 | 190.75 | 407.50 | 1273.00 | 5357.00 |
| FC | 1.00 | 252.75 | 452.00 | 1679.00 | 5441.00 |
| RP | 1.00 | 181.75 | 393.00 | 877.75 | 4528.00 |
| modT | 44.00 | 264.75 | 1834.00 | 4708.25 | 11771.00 |
| samT | 44.00 | 264.75 | 1858.50 | 4834.25 | 12217.00 |
| shrinkT | 44.00 | 262.25 | 1758.00 | 4359.25 | 10487.00 |
| ibmT | 44.00 | 263.25 | 1811.00 | 4635.50 | 11743.00 |

**8-2.**

| Method | MAS | RMA | DFW |
| --- | --- | --- | --- |
| *w* | 0.85881 | 0.85553 | 0.81322 |
| AD | 0.83396 | 0.92172 | 0.94666 |
| WAD | 0.93599 | 0.94599 | 0.95017 |
| FC | 0.83954 | 0.92057 | 0.94666 |
| RP | 0.77378 | 0.93873 | 0.96208 |
| modT | 0.92496 | 0.89196 | 0.85950 |
| samT | 0.88416 | 0.87358 | 0.85531 |
| shrinkT | 0.92591 | 0.86567 | 0.87144 |
| ibmT | 0.94547 | 0.89584 | 0.86085 |

**Dataset 33**

**1.** http://www.ncbi.nlm.nih.gov/sites/entrez?Db=Pubmed&term=16358311[UID]

**2.** GSE1577

**3.** 9 T-LL samples:

GSM27065-27066, GSM27068-27069, GSM27071-27072,

GSM27074-27075, GSM27077

**4.** 10 T-ALL samples:

GSM27079, GSM27082-27083, GSM27085, GSM27087-27088,

GSM27091, GSM27093-27095

**5.** 9 probesets.

"201162_at", "201163_s_at", "201667_at", "210495_x_at", "211719_x_at",

"212464_s_at", "216442_x_at", "211997_x_at", "211998_at"

**6.** MAS5.0

**7.** RMA- and DFW-preprocessed data were obtained from a total of **29** samples in GSE1577.

**8-1.**

| 8-1. Percentile ranks for true positives (TPs) | | | |  |  |
| --- | --- | --- | --- | --- | --- |
| MAS-preprocessed data | | |  |  |  |
| Method | 0% | 25% | 50% | 75% | 100% |
| *w* | 791.00 | 2209.00 | 6516.00 | 8040.00 | 12304.00 |
| AD | 1.00 | 7.00 | 10.00 | 55.00 | 1863.00 |
| WAD | 4.00 | 7.00 | 11.00 | 17.00 | 533.00 |
| FC | 1.00 | 9.00 | 27.00 | 113.00 | 1654.00 |
| RP | 1.00 | 8.00 | 14.00 | 59.00 | 1596.00 |
| modT | 3.00 | 11.00 | 33.00 | 71.00 | 399.00 |
| samT | 1.00 | 9.00 | 17.00 | 41.00 | 669.00 |
| shrinkT | 2.00 | 12.00 | 32.00 | 62.00 | 448.00 |
| ibmT | 3.00 | 7.00 | 28.00 | 67.00 | 328.00 |
| RMA-preprocessed data | | |  |  |  |
| Method | 0% | 25% | 50% | 75% | 100% |
| *w* | 752.00 | 1591.00 | 3635.00 | 5031.00 | 12084.00 |
| AD | 5.00 | 14.00 | 21.00 | 24.00 | 650.00 |
| WAD | 4.00 | 14.00 | 26.00 | 31.00 | 251.00 |
| FC | 3.00 | 8.00 | 18.00 | 33.00 | 859.00 |
| RP | 5.00 | 12.00 | 21.00 | 29.00 | 467.00 |
| modT | 11.00 | 37.00 | 74.00 | 106.00 | 226.00 |
| samT | 10.00 | 25.00 | 56.00 | 82.00 | 171.00 |
| shrinkT | 11.00 | 32.00 | 67.00 | 91.00 | 207.00 |
| ibmT | 10.00 | 28.00 | 61.00 | 112.00 | 181.00 |
| DFW-preprocessed data | | |  |  |  |
| Method | 0% | 25% | 50% | 75% | 100% |
| *w* | 554.00 | 2307.00 | 3772.00 | 5777.00 | 20748.00 |
| AD | 11.00 | 18.00 | 44.00 | 62.00 | 303.00 |
| WAD | 9.00 | 35.00 | 56.00 | 94.00 | 844.00 |
| FC | 11.00 | 17.00 | 43.00 | 67.00 | 302.00 |
| RP | 5.00 | 15.00 | 24.00 | 30.00 | 177.00 |
| modT | 6.00 | 33.00 | 269.00 | 472.00 | 663.00 |
| samT | 6.00 | 33.00 | 265.00 | 455.00 | 642.00 |
| shrinkT | 6.00 | 32.00 | 239.00 | 403.00 | 549.00 |
| ibmT | 6.00 | 33.00 | 267.00 | 472.00 | 662.00 |

**8-2.**

| Method | MAS | RMA | DFW |
| --- | --- | --- | --- |
| *w* | 0.75084 | 0.81213 | 0.73529 |
| AD | 0.98983 | 0.99619 | 0.99651 |
| WAD | 0.99707 | 0.99741 | 0.99373 |
| FC | 0.99014 | 0.99496 | 0.99648 |
| RP | 0.99124 | 0.99704 | 0.99806 |
| modT | 0.99638 | 0.99643 | 0.98820 |
| samT | 0.99582 | 0.99729 | 0.98854 |
| shrinkT | 0.99635 | 0.99676 | 0.98989 |
| ibmT | 0.99692 | 0.99691 | 0.98822 |

**Dataset 34**

**1.** http://www.ncbi.nlm.nih.gov/sites/entrez?Db=Pubmed&term=15877233[UID]

**2.** GSE2240

**3.** 17 atrial appendages:

GSM40991-41005, GSM41008-41009

**4.** 5 control samples:

GSM41010-41014

**5.** 3 probesets.

"210239_at", "203997_at", "205508_at"

**6.** MAS5.0

**7.** RMA- and DFW-preprocessed data were obtained from a total of **22** samples in GSE2240.

**8-1.**

| 8-1. Percentile ranks for true positives (TPs) | | | |  |  |
| --- | --- | --- | --- | --- | --- |
| MAS-preprocessed data | | |  |  |  |
| Method | 0% | 25% | 50% | 75% | 100% |
| *w* | 2959.00 | 4704.00 | 6449.00 | 8400.50 | 10352.00 |
| AD | 112.00 | 120.00 | 128.00 | 2715.50 | 5303.00 |
| WAD | 36.00 | 83.00 | 130.00 | 1910.50 | 3691.00 |
| FC | 121.00 | 137.50 | 154.00 | 2261.00 | 4368.00 |
| RP | 67.00 | 69.00 | 71.00 | 3878.00 | 7685.00 |
| modT | 22.00 | 250.00 | 478.00 | 2658.50 | 4839.00 |
| samT | 38.00 | 89.50 | 141.00 | 2328.50 | 4516.00 |
| shrinkT | 21.00 | 201.50 | 382.00 | 2519.00 | 4656.00 |
| ibmT | 20.00 | 270.50 | 521.00 | 2515.00 | 4509.00 |
| RMA-preprocessed data | | |  |  |  |
| Method | 0% | 25% | 50% | 75% | 100% |
| *w* | 3012.00 | 3859.00 | 4706.00 | 8775.00 | 12844.00 |
| AD | 39.00 | 66.00 | 93.00 | 1182.00 | 2271.00 |
| WAD | 31.00 | 213.00 | 395.00 | 1269.50 | 2144.00 |
| FC | 41.00 | 72.00 | 103.00 | 1155.00 | 2207.00 |
| RP | 18.00 | 29.50 | 41.00 | 1242.50 | 2444.00 |
| modT | 29.00 | 41.00 | 53.00 | 2294.50 | 4536.00 |
| samT | 26.00 | 38.50 | 51.00 | 2192.00 | 4333.00 |
| shrinkT | 28.00 | 40.50 | 53.00 | 2343.00 | 4633.00 |
| ibmT | 29.00 | 40.00 | 51.00 | 2318.00 | 4585.00 |
| DFW-preprocessed data | | |  |  |  |
| Method | 0% | 25% | 50% | 75% | 100% |
| *w* | 6874.00 | 6888.50 | 6903.00 | 11920.50 | 16938.00 |
| AD | 70.00 | 187.00 | 304.00 | 3538.50 | 6773.00 |
| WAD | 70.00 | 265.50 | 461.00 | 3406.50 | 6352.00 |
| FC | 71.00 | 188.50 | 306.00 | 3539.00 | 6772.00 |
| RP | 16.00 | 59.00 | 102.00 | 4216.50 | 8331.00 |
| modT | 26.00 | 45.50 | 65.00 | 7377.50 | 14690.00 |
| samT | 26.00 | 45.50 | 65.00 | 7357.50 | 14650.00 |
| shrinkT | 26.00 | 45.00 | 64.00 | 6269.50 | 12475.00 |
| ibmT | 26.00 | 45.50 | 65.00 | 7357.50 | 14650.00 |

**8-2.**

| Method | MAS | RMA | DFW |
| --- | --- | --- | --- |
| *w* | 0.70446 | 0.69246 | 0.54056 |
| AD | 0.91716 | 0.96414 | 0.89316 |
| WAD | 0.94238 | 0.96164 | 0.89711 |
| FC | 0.93063 | 0.96492 | 0.89313 |
| RP | 0.88305 | 0.96264 | 0.87368 |
| modT | 0.92021 | 0.93100 | 0.77895 |
| samT | 0.92985 | 0.93411 | 0.77955 |
| shrinkT | 0.92440 | 0.92956 | 0.81210 |
| ibmT | 0.92454 | 0.93030 | 0.77955 |

**Dataset 35**

**1.** http://www.ncbi.nlm.nih.gov/sites/entrez?Db=Pubmed&term=15817885[UID]

**2.** GSE2240

**3.** 10 atrial fibrillation samples:

GSM40980-40989

**4.** 20 sinus rhythm samples:

GSM40990-41009

**5.** 9 probesets.

"214316_x_at", "204312_x_at", "202765_s_at", "208351_s_at", "210555_s_at",

"209239_at", "205026_at", "215253_s_at", "213453_x_at"

**6.** RMA

**7.** DFW-preprocessed data were obtained from a total of **35** samples in GSE2240.

**8-1.**

| 8-1. Percentile ranks for true positives (TPs) | | | |  |  |
| --- | --- | --- | --- | --- | --- |
| MAS-preprocessed data | | |  |  |  |
| Method | 0% | 25% | 50% | 75% | 100% |
| *w* | 33.00 | 5016.00 | 8017.00 | 8847.00 | 13002.00 |
| AD | 912.00 | 1616.00 | 6135.00 | 12983.00 | 17673.00 |
| WAD | 413.00 | 825.00 | 4211.00 | 6158.00 | 16180.00 |
| FC | 1173.00 | 2074.00 | 5807.00 | 12351.00 | 17373.00 |
| RP | 1660.00 | 2429.00 | 9682.00 | 17203.00 | 19818.00 |
| modT | 378.00 | 912.00 | 1235.00 | 5519.00 | 15545.00 |
| samT | 316.00 | 951.00 | 1337.00 | 7856.00 | 16276.00 |
| shrinkT | 344.00 | 870.00 | 1352.00 | 6147.00 | 15696.00 |
| ibmT | 367.00 | 813.00 | 1382.00 | 4470.00 | 15290.00 |
| RMA-preprocessed data | | |  |  |  |
| Method | 0% | 25% | 50% | 75% | 100% |
| *w* | 20.00 | 4365.00 | 5896.00 | 9913.00 | 14120.00 |
| AD | 217.00 | 540.00 | 1206.00 | 2905.00 | 8309.00 |
| WAD | 289.00 | 569.00 | 1810.00 | 2157.00 | 7106.00 |
| FC | 267.00 | 554.00 | 1288.00 | 2900.00 | 8400.00 |
| RP | 245.00 | 608.00 | 1330.00 | 4141.00 | 12337.00 |
| modT | 13.00 | 200.00 | 594.00 | 1774.00 | 2556.00 |
| samT | 15.00 | 163.00 | 539.00 | 1842.00 | 2768.00 |
| shrinkT | 13.00 | 205.00 | 613.00 | 1774.00 | 2523.00 |
| ibmT | 14.00 | 188.00 | 589.00 | 1912.00 | 2649.00 |
| DFW-preprocessed data | | |  |  |  |
| Method | 0% | 25% | 50% | 75% | 100% |
| *w* | 6.00 | 6946.00 | 9235.00 | 10069.00 | 18533.00 |
| AD | 615.00 | 2102.00 | 3107.00 | 4598.00 | 12133.00 |
| WAD | 647.00 | 2488.00 | 3424.00 | 4927.00 | 11501.00 |
| FC | 613.00 | 2101.00 | 3106.00 | 4597.00 | 12133.00 |
| RP | 462.00 | 1855.00 | 2673.00 | 4295.00 | 13661.00 |
| modT | 42.00 | 330.00 | 499.00 | 539.00 | 1937.00 |
| samT | 42.00 | 317.00 | 491.00 | 560.00 | 2112.00 |
| shrinkT | 42.00 | 262.00 | 409.00 | 1303.00 | 4194.00 |
| ibmT | 43.00 | 331.00 | 462.00 | 537.00 | 2589.00 |

**8-2.**

| Method | MAS | RMA | DFW |
| --- | --- | --- | --- |
| *w* | 0.67380 | 0.67698 | 0.62253 |
| AD | 0.67150 | 0.89571 | 0.79997 |
| WAD | 0.74978 | 0.91150 | 0.81383 |
| FC | 0.67447 | 0.89415 | 0.80001 |
| RP | 0.57026 | 0.85938 | 0.80704 |
| modT | 0.80552 | 0.95591 | 0.97000 |
| samT | 0.77906 | 0.95273 | 0.96951 |
| shrinkT | 0.80011 | 0.95679 | 0.95812 |
| ibmT | 0.80635 | 0.95367 | 0.96688 |

**Dataset 36**

**1.** http://www.ncbi.nlm.nih.gov/sites/entrez?Db=Pubmed&term=16797695[UID]

**2.** GSE2531

**3.** 3 JEG3 samples:

GSM48266-48268

**4.** 4 BeWo samples:

GSM48269-48272

**5.** 17 probesets.

"202910_s_at", "204271_s_at", "204273_at", "206701_x_at", "201910_at",

"201911_s_at", "201562_s_at", "201563_at", "203665_at", "203510_at",

"211599_x_at", "213807_x_at", "213816_s_at", "205322_s_at", "205323_s_at",

"201286_at", "201287_s_at"

**6.** RMA

**7.** DFW-preprocessed data were obtained from a total of **7** samples in GSE2531.

**8-1.**

| 8-1. Percentile ranks for true positives (TPs) | | | |  |  |
| --- | --- | --- | --- | --- | --- |
| MAS-preprocessed data | | |  |  |  |
| Method | 0% | 25% | 50% | 75% | 100% |
| *w* | 4023.00 | 7584.00 | 8659.00 | 10812.00 | 16109.00 |
| AD | 4.00 | 148.00 | 670.00 | 2825.00 | 5137.00 |
| WAD | 18.00 | 170.00 | 323.00 | 2186.00 | 7165.00 |
| FC | 7.00 | 135.00 | 604.00 | 2586.00 | 7192.00 |
| RP | 4.00 | 174.00 | 476.00 | 1885.00 | 6702.00 |
| modT | 9.00 | 43.00 | 370.00 | 957.00 | 12339.00 |
| samT | 29.00 | 69.00 | 382.00 | 1738.00 | 7662.00 |
| shrinkT | 11.00 | 43.00 | 363.00 | 981.00 | 12150.00 |
| ibmT | 9.00 | 152.00 | 352.00 | 1286.00 | 11794.00 |
| RMA-preprocessed data | | |  |  |  |
| Method | 0% | 25% | 50% | 75% | 100% |
| *w* | 3262.00 | 7017.00 | 8897.00 | 11614.00 | 18163.00 |
| AD | 20.00 | 112.00 | 433.00 | 2559.00 | 8985.00 |
| WAD | 12.00 | 338.00 | 632.00 | 3196.00 | 12566.00 |
| FC | 19.00 | 105.00 | 395.00 | 2564.00 | 8968.00 |
| RP | 18.00 | 108.00 | 440.00 | 2464.00 | 9625.00 |
| modT | 33.00 | 142.00 | 649.00 | 1227.00 | 7225.00 |
| samT | 55.00 | 131.00 | 781.00 | 1336.00 | 6714.00 |
| shrinkT | 33.00 | 142.00 | 646.00 | 1227.00 | 7222.00 |
| ibmT | 30.00 | 136.00 | 792.00 | 1318.00 | 6705.00 |
| DFW-preprocessed data | | |  |  |  |
| Method | 0% | 25% | 50% | 75% | 100% |
| *w* | 5265.00 | 7536.00 | 11553.00 | 16379.00 | 22265.00 |
| AD | 9.00 | 149.00 | 577.00 | 3638.00 | 10150.00 |
| WAD | 10.00 | 404.00 | 964.00 | 4074.00 | 11669.00 |
| FC | 9.00 | 149.00 | 577.00 | 3638.00 | 10150.00 |
| RP | 9.00 | 150.00 | 601.00 | 3573.00 | 9888.00 |
| modT | 51.00 | 291.00 | 1522.00 | 2317.00 | 8899.00 |
| samT | 52.00 | 295.00 | 1562.00 | 2379.00 | 9400.00 |
| shrinkT | 49.00 | 281.00 | 1421.00 | 2142.00 | 8167.00 |
| ibmT | 50.00 | 285.00 | 1499.00 | 2272.00 | 9046.00 |

**8-2.**

| Method | MAS | RMA | DFW |
| --- | --- | --- | --- |
| *w* | 0.58008 | 0.56549 | 0.44764 |
| AD | 0.93468 | 0.91997 | 0.88989 |
| WAD | 0.94139 | 0.89755 | 0.87748 |
| FC | 0.93211 | 0.91981 | 0.88989 |
| RP | 0.93460 | 0.92189 | 0.89917 |
| modT | 0.94200 | 0.94304 | 0.91469 |
| samT | 0.94468 | 0.94577 | 0.91184 |
| shrinkT | 0.94269 | 0.94306 | 0.91672 |
| ibmT | 0.93799 | 0.94212 | 0.91561 |

**Dataset 37**

**1.** http://www.ncbi.nlm.nih.gov/sites/entrez?Db=Pubmed&term=16894394[UID]

**2.** GSE5389

**3.**10 bipolar samples:

GSM123243-123252

**4.** 11 control samples:

GSM123253-123263

**5.** 6 probesets.

"202346_at", "201133_s_at", "201672_s_at", "204313_s_at", "212216_at",

"203998_s_at"

**6.** RMA

**7.** DFW-preprocessed data were obtained from a total of 21 samples in GSE5389.

**8-1.**

| 8-1. Percentile ranks for true positives (TPs) | | | |  |  |
| --- | --- | --- | --- | --- | --- |
| MAS-preprocessed data | | |  |  |  |
| Method | 0% | 25% | 50% | 75% | 100% |
| *w* | 781.00 | 1924.00 | 2340.00 | 4973.75 | 7502.00 |
| AD | 6.00 | 1129.50 | 1586.50 | 2345.00 | 6322.00 |
| WAD | 1.00 | 244.75 | 324.50 | 449.25 | 2482.00 |
| FC | 263.00 | 1736.50 | 2248.00 | 2384.50 | 7040.00 |
| RP | 19.00 | 2518.75 | 2783.00 | 4503.00 | 9706.00 |
| modT | 2.00 | 376.25 | 958.00 | 1204.50 | 1278.00 |
| samT | 17.00 | 268.00 | 555.00 | 989.75 | 2005.00 |
| shrinkT | 14.00 | 308.50 | 726.00 | 1029.50 | 1651.00 |
| ibmT | 1.00 | 278.50 | 737.50 | 937.00 | 1017.00 |
| RMA-preprocessed data | | |  |  |  |
| Method | 0% | 25% | 50% | 75% | 100% |
| *w* | 966.00 | 1897.00 | 2900.50 | 7173.25 | 10102.00 |
| AD | 1.00 | 111.25 | 264.00 | 319.25 | 753.00 |
| WAD | 2.00 | 69.00 | 369.00 | 714.75 | 859.00 |
| FC | 11.00 | 167.75 | 368.00 | 413.75 | 884.00 |
| RP | 1.00 | 133.75 | 362.00 | 443.25 | 1126.00 |
| modT | 2.00 | 332.25 | 391.00 | 457.25 | 773.00 |
| samT | 2.00 | 213.50 | 318.00 | 355.75 | 621.00 |
| shrinkT | 2.00 | 299.75 | 354.00 | 413.50 | 714.00 |
| ibmT | 4.00 | 280.00 | 374.50 | 394.75 | 657.00 |
| DFW-preprocessed data | | |  |  |  |
| Method | 0% | 25% | 50% | 75% | 100% |
| *w* | 1239.00 | 3003.00 | 7720.00 | 12290.75 | 15127.00 |
| AD | 10.00 | 295.75 | 1243.50 | 1966.25 | 2230.00 |
| WAD | 24.00 | 258.50 | 1166.50 | 2024.25 | 2817.00 |
| FC | 14.00 | 294.75 | 1243.00 | 1967.00 | 2235.00 |
| RP | 3.00 | 144.75 | 902.00 | 1978.75 | 2170.00 |
| modT | 5.00 | 455.25 | 676.00 | 1197.50 | 1590.00 |
| samT | 4.00 | 405.50 | 627.00 | 1142.50 | 1492.00 |
| shrinkT | 2.00 | 249.75 | 390.50 | 727.75 | 975.00 |
| ibmT | 4.00 | 441.50 | 659.50 | 1174.50 | 1564.00 |

**8-2.**

| Method | MAS | RMA | DFW |
| --- | --- | --- | --- |
| *w* | 0.84604 | 0.79794 | 0.64814 |
| AD | 0.90233 | 0.98759 | 0.94843 |
| WAD | 0.97142 | 0.98222 | 0.94449 |
| FC | 0.88199 | 0.98399 | 0.94837 |
| RP | 0.82959 | 0.98236 | 0.95372 |
| modT | 0.96526 | 0.98258 | 0.96499 |
| samT | 0.96695 | 0.98665 | 0.96727 |
| shrinkT | 0.96711 | 0.98415 | 0.97920 |
| ibmT | 0.97306 | 0.98475 | 0.96573 |

**Dataset 38**

**1.** http://www.ncbi.nlm.nih.gov/sites/entrez?Db=Pubmed&term=17950572[UID]

**2.** GSE5390

**3.** 7 down syndrome samples:

GSM123264-123270

**4.** 8 control samples:

GSM123271-123278

**5.** 8 probesets.

"222162_s_at", "217867_x_at", "208370_s_at", "209033_s_at", "200677_at",

"209686_at", "203381_s_at", "212377_s_at"

**6.** RMA

**7.** DFW-preprocessed data were obtained from a total of 15 samples in GSE5390.

**8-1.**

| 8-1. Percentile ranks for true positives (TPs) | | | |  |  |
| --- | --- | --- | --- | --- | --- |
| MAS-preprocessed data | | |  |  |  |
| Method | 0% | 25% | 50% | 75% | 100% |
| *w* | 1078.00 | 1339.50 | 2223.00 | 4597.00 | 10702.00 |
| AD | 126.00 | 426.50 | 1047.00 | 2937.50 | 6572.00 |
| WAD | 29.00 | 54.00 | 467.50 | 1032.25 | 3068.00 |
| FC | 158.00 | 526.75 | 943.00 | 2915.50 | 6016.00 |
| RP | 143.00 | 509.00 | 1218.50 | 3265.50 | 7400.00 |
| modT | 4.00 | 176.00 | 426.50 | 644.00 | 1621.00 |
| samT | 4.00 | 183.50 | 331.00 | 814.25 | 2031.00 |
| shrinkT | 4.00 | 163.25 | 359.00 | 718.25 | 1763.00 |
| ibmT | 2.00 | 112.25 | 355.00 | 481.00 | 1204.00 |
| RMA-preprocessed data | | |  |  |  |
| Method | 0% | 25% | 50% | 75% | 100% |
| *w* | 954.00 | 1356.75 | 2551.50 | 5691.25 | 8598.00 |
| AD | 17.00 | 88.25 | 288.00 | 1009.25 | 1304.00 |
| WAD | 17.00 | 47.50 | 322.50 | 611.25 | 1930.00 |
| FC | 32.00 | 127.75 | 321.00 | 989.75 | 1204.00 |
| RP | 27.00 | 105.50 | 359.00 | 1142.50 | 1545.00 |
| modT | 7.00 | 173.00 | 344.50 | 630.25 | 1497.00 |
| samT | 7.00 | 153.75 | 340.00 | 566.50 | 1539.00 |
| shrinkT | 6.00 | 136.50 | 298.50 | 508.75 | 1325.00 |
| ibmT | 5.00 | 165.75 | 393.50 | 534.00 | 1425.00 |
| DFW-preprocessed data | | |  |  |  |
| Method | 0% | 25% | 50% | 75% | 100% |
| *w* | 1170.00 | 1652.50 | 2600.00 | 7118.25 | 10273.00 |
| AD | 5.00 | 57.25 | 544.50 | 1587.50 | 3842.00 |
| WAD | 8.00 | 37.00 | 559.00 | 1316.25 | 4283.00 |
| FC | 5.00 | 56.75 | 544.00 | 1587.25 | 3843.00 |
| RP | 3.00 | 45.25 | 546.50 | 1282.25 | 4989.00 |
| modT | 16.00 | 257.50 | 683.00 | 1068.50 | 7762.00 |
| samT | 18.00 | 278.25 | 724.50 | 1118.50 | 7983.00 |
| shrinkT | 9.00 | 143.00 | 381.00 | 628.75 | 5432.00 |
| ibmT | 18.00 | 267.25 | 703.50 | 1099.25 | 7814.00 |

**8-2.**

| Method | MAS | RMA | DFW |
| --- | --- | --- | --- |
| *w* | 0.84288 | 0.83660 | 0.80088 |
| AD | 0.90501 | 0.97680 | 0.95409 |
| WAD | 0.96345 | 0.97749 | 0.95442 |
| FC | 0.90949 | 0.97684 | 0.95410 |
| RP | 0.89352 | 0.97308 | 0.95074 |
| modT | 0.97626 | 0.97855 | 0.93452 |
| samT | 0.97203 | 0.97904 | 0.93206 |
| shrinkT | 0.97464 | 0.98170 | 0.95697 |
| ibmT | 0.98214 | 0.97957 | 0.93356 |
